# Seeing through arthropod eyes: An AI-assisted, biomimetic approach for high-resolution, multi-task imaging

**DOI:** 10.1126/sciadv.adt3505

**Published:** 2025-05-21

**Authors:** Yan Long, Bo Dai, Chenliang Chang, Neil Upreti, Li Wei, Lulu Zheng, Songlin Zhuang, Tony Jun Huang, Dawei Zhang

**Affiliations:** ^1^ Engineering Research Center of Optical Instrument and System, the Ministry of Education, Shanghai Key Laboratory of Modern Optical System, University of Shanghai for Science and Technology, Shanghai 200093, China.; ^2^ Department of Mechanical Engineering and Materials Science, Duke University, Durham, NC 27709, USA.

## Abstract

Arthropods have intricate compound eyes and optic neuropils, exhibiting exceptional visual capabilities. Combining the strengths of digital imaging with the features of natural arthropod visual systems offers a promising approach to harness wide-angle vision and depth perception while addressing limitations like low resolving power. Here, we present an artificial intelligence–assisted biomimetic system modeled after arthropod vision. We developed a biomimetic compound eye camera with an effective pixel number of 4.3 megapixels capable of producing full-color panoramic images with a viewing angle of 165° and resolving power of 40 micrometers. Using rich visual information, our system achieves high-fidelity image reconstruction, precise 3D position prediction, high-accuracy classification, and pattern recognition through a multistage neural network. Moreover, our compact biomimetic visual system can simultaneously track the 3D motion of multiple miniature targets independently. The proof-of-concept biomimetic arthropod visual system offers a computational panoramic imaging solution, advancing applications in industry, medicine, and robotics.

## Teaser

AI-assisted biomimetic arthropod visual system enables full-color high-resolution panoramic imaging and visual cognition.

## INTRODUCTION

**Fig. 1.**
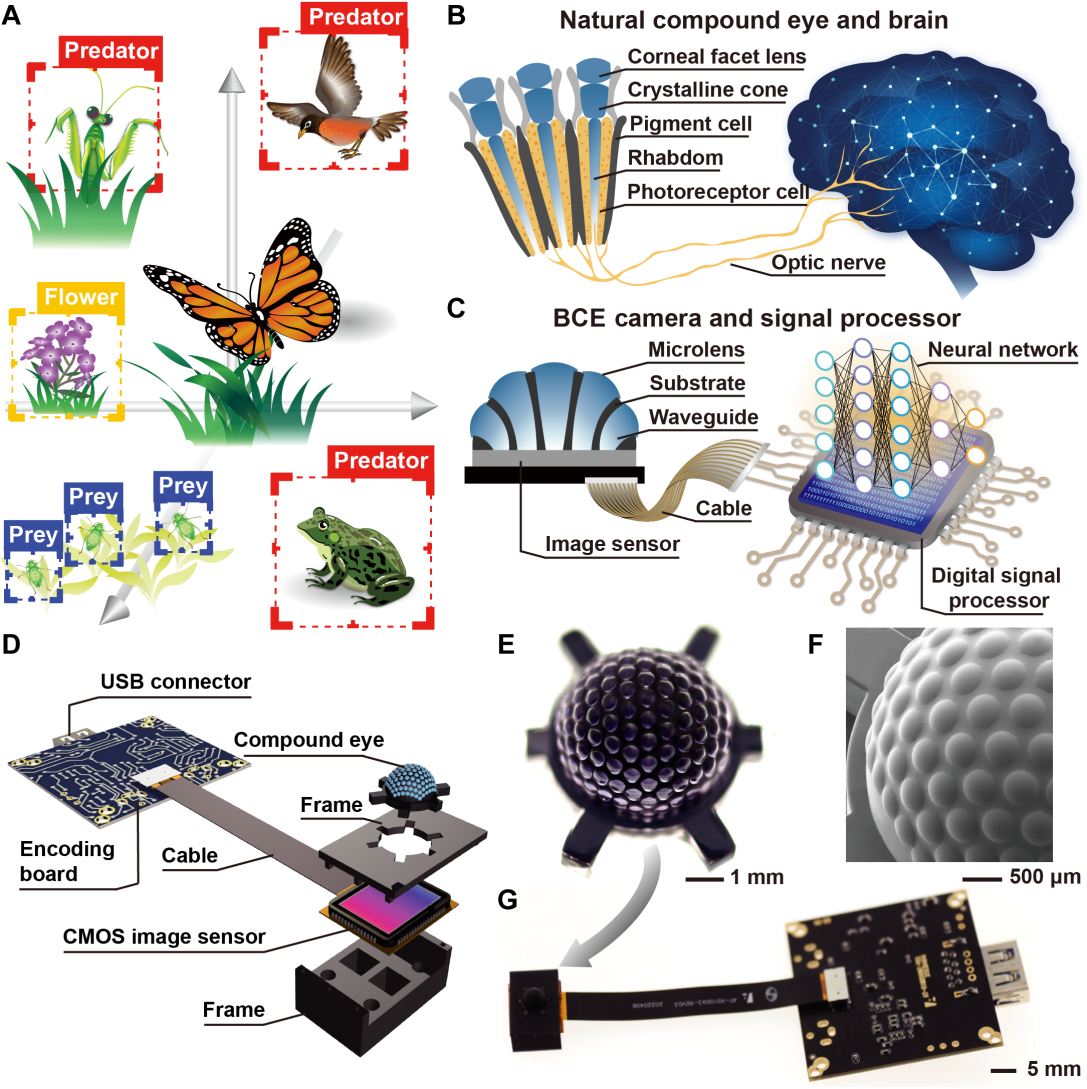
BCE and artificial visual system. (**A**) Scenario of an arthropod surrounded by predators and prey. (**B**) Illustration of a natural visual system consisting of a compound eye and a brain. (**C**) Illustration of an artificial visual system consisting of the BCE camera and digital signal processor. (**D**) Exploded view of the BCE camera. (**E**) Image of the BCE. CMOS, complementary metal-oxide semiconductor. (**F**) Scanning electron microscopy image of the BCE. (**G**) Image of the BCE camera.

A visual system is composed of eyes and nervous systems that sense physical stimuli and interpret information into a mental representation of the world. Arthropod visual systems have evolved over more than 500 million years, dating back to the Cambrian era (*1*, *2*). The elaborate structure of compound eyes is a remarkable product optimized through evolution. A compound eye with hundreds and thousands of ommatidia can detect light from different directions, providing a wide-angle field of view and enabling depth perception. However, each ommatidium contains only a few photoreceptor cells. In some amphipods, up to 11 photoreceptor cells per ommatidium have been observed (*3*, *4*). Arthropods, such as amphipods and butterflies, with large compound eyes and numerous ommatidia, exhibit mosaic-like vision with very low spatial resolution (*5*). Despite their subpar vision quality and weak brain power, arthropods display remarkable visual cognitive abilities, as evidenced by complex visual learning behaviors such as color learning, pattern recognition, and visual attention seen in social insects (*6*). Arthropods can accurately identify predators, prey, food sources, and host plants based on the chromatic and geometric cues collected by their visual systems, as illustrated in Fig. 1A.

The compound eye is an essential sensory organ in the arthropod visual system, attracting significant research interest in the context of biomimetic eye development (*7*–*15*). However, matching a hemispherical artificial compound eye with a sensing device to omnidirectionally collect signals has been a great challenge (*16*–*18*). One strategy involves integrating a flexible photodetector array (*19*–*22*). Each ommatidium detects light intensity at a single pixel along a specific orientation, and the compound eye generates a pixelated image. Rather than developing flexible optoelectronics for the compound eye, another strategy is to guide the light collected by the compound eye to a planar image sensor. Engineering microlenses with a logarithmic profile could extend the focus range, allowing the compound eye to directly focus images with a limited angle of view onto the planar image sensor (*23*). In addition, mimicking the anatomical structure of an apposition compound eye enables efficient, omnidirectional light collection, which can then be directed to an underlying planar surface (*24*). The biomimetic compound eye (BCE) shares the same limitations as its natural counterpart: The tiny ommatidium covers a small portion of the pixel area, lacks comprehensive imaging capabilities, and only contributes a few pixels to the image, resulting in a mosaic-like image. As a result, the imaging quality of the BCE still requires significant improvement.

Beyond BCEs, visual cognition has been explored by some pioneering work for artificial visual systems (*25*–*29*). While their imaging performance remains limited, these dedicated visual systems are capable of handling specific tasks such as motion sensing, depth perception, and collision detection. For instance, to detect collisions, a BCE using a threshold-switching memristor array that functions as a lobula giant movement detector was developed (*30*). For depth perception, a visual system that integrated a photodetector, a memristive spiking encoder and floating-gate synaptic transistors was presented (*31*). Unlike these specialized systems, visual systems capable of producing high-quality images have the potential to perform diverse tasks.

Here, we demonstrate a biomimetic arthropod visual system that harnesses artificial intelligence to enable a BCE camera to intelligently detect, track, and recognize multiple targets with a wide angle of view in a cramped area. The compact, lightweight BCE camera has a wide viewing angle of over 165° × 360° (polar angle × azimuthal angle) and an effective pixel number of 4.3 megapixels. High-quality, full-color panoramic imaging can be achieved with a resolving power of 40 μm. In addition to its extraordinary imaging capability, the system also features impressive visual cognition capabilities, including three-dimensional (3D) position prediction, color classification, and pattern recognition. The developed biomimetic arthropod visual system paves an avenue for research and applications in computational multi-ocular systems for stereo vision, motion tracking, and endoscopic diagnosis.

## RESULTS

### BCE camera

Natural compound eyes, found in most diurnal insects, have a sophisticated anatomical structure. A compound eye consists of numerous ommatidia, each detecting light from different directions (Fig. 1A). Light is collected by a facet lens and directed into a rhabdom via a crystalline cone. Photoreceptor cells surrounding the rhabdom convert the light into electrical signals that are transmitted to the brain via optic nerve, as shown in Fig. 1B. The details of the BCE structure and fabrication process are provided in Materials and Methods and figs. S1 to S4. A microlens array is positioned on the surface of a hemispherical substrate, as shown in Fig. 1C. Optical waveguides link the microlenses to the flat base of the hemisphere. Each ommatidium, consisting of a microlens and an optical waveguide, can guide the incident light from a certain range onto the flat base. Thus, the biomimetic eye can be directly integrated into the planar image sensor, forming a BCE camera, as shown in Fig. 1D.

The BCE-camera imaging process is straightforward, achieved by averaging the light intensity from each ommatidium (*24*). Each ommatidium contributes one pixel to the resulting panoramic image. However, the pixel number is limited because the number of ommatidia is constrained by the resolution of 3D printing. In addition, the small size of each ommatidium, which is inversely proportional to the number of ommatidia, has a low detection efficiency because the small aperture restricts the amount of incident light and causes multiple internal reflections in the narrow waveguide, leading to high optical loss (fig. S5). An alternative strategy for imaging is to exploit ommatidia as an imager array. The images captured by the adjacent ommatidia are further processed to form a high-quality image. In this strategy, BCE designs with larger ommatidia are preferred, as ommatidia’s ability to form clear images on the image sensor can be enhanced because of higher detection efficiency.

Figure 1 (E and F) illustrates the BCE with relatively large ommatidia. The BCE features a hemispherical top and a thin pedestal designed for integration with the image sensor. The BCE has a diameter of 5 mm and contains 127 ommatidia (details in Materials and Methods). The ommatidia are oriented at polar angles, α, ranging from 0° to 82.5°, and azimuthal angle, β, ranging from 0° to 360°. Each ommatidium consists of a microlens on a corresponding cylindrical optical waveguide. The microlenses are distributed omnidirectionally on the hemisphere’s surface. The diameter and curvature radius of the microlenses are 455 and 667 μm, respectively. The design of the microlens is explained in Materials and Methods. The optical waveguides narrow down from the hemispherical surface (*d*_T_ = 450 μm) to the flat base of the BCE (*d*_B_ = 320 μm). The optical waveguide outputs are hexagonally arranged at the base. The separation between the proximal ends of the waveguides, *d*_S_, is 60 μm. This feature size of the structure is constrained by the resolution of 3D printing.

The BCE is mounted within a frame and assembled onto a commercially available color image sensor. The frame aligns the BCE with the image sensor and blocks ambient light. The image sensor directly detects the output from the BCE. The BCE camera has a compact size of 15 mm by 10 mm by 5.3 mm. The pixel density of the image sensor and the diameter of the proximal end of the waveguide determine the effective pixel number per ommatidium. The image sensor with a small pixel size (1.55 μm by 1.55 μm) is adopted. Every ommatidium can contribute a sub-image of 33,475 pixels. The total number of effective pixels in the BCE camera that are physically involved in the imaging is 4.3 megapixels. The images obtained by the BCE camera can provide a wealth of information for visual cognition.

### Characterization of the BCE camera

**Fig. 2.**
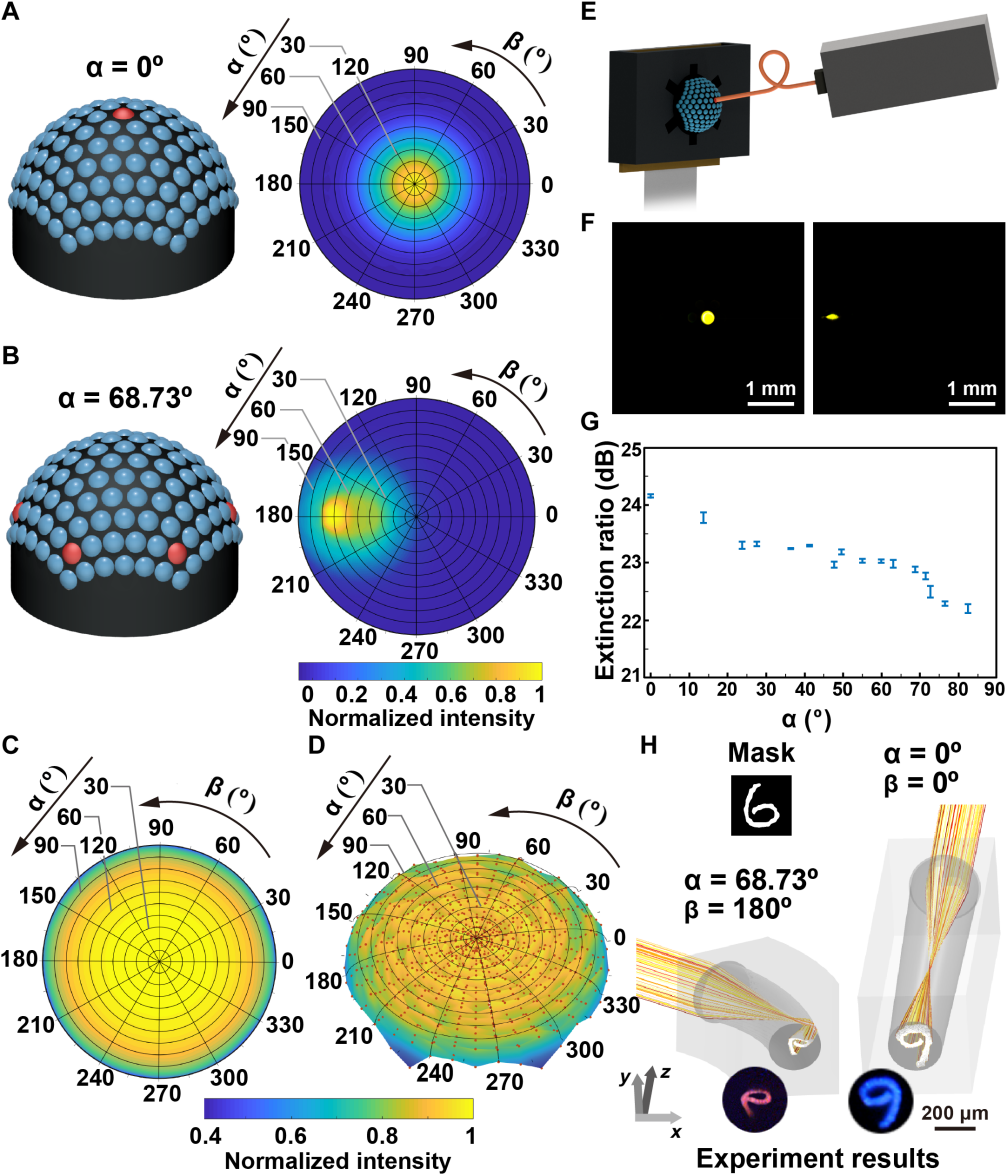
Optical characterization of the BCE. (**A**) The ASF of the central ommatidium (α = 0°, β = 0°). (**B**) The ASF of the ommatidia with the polar angle of 68.73°. (**C**) The ASF of the BCE. (**D**) Measurement of the ASF for the BCE. The ASF was estimated by measuring the response of the ommatidia under the illumination of the collimated light with different incident angles. Each data point stands for the summation of the measured light intensity for the incidence from a specific direction. The surface is generated by interpolating the scattered data points. (**E**) Experiment setup for measuring the optical properties of the ommatidia. (**F**) The intensity distribution at the proximal ends of the optical waveguides of the ommatidia with the orientations of (α = 0°, β = 0°) and (α = 68.73°, β = 180°). (**G**) Evaluation of the optical cross-talk among the ommatidia. (**H**) Simulation of the light propagation in the ommatidia and the images captured at the proximal ends of the optical waveguides.

To study the optical properties of the BCE camera, we first analyzed its angular sensitivity. In the simulation, collimated light illuminated the ommatidia with different incident angles (α*′* or β*′*) as depicted in fig. S6. The angular sensitivity function (ASF) of the ommatidium was determined by calculating the output of the ommatidium as a function of the incident angle. The ASF of the central ommatidium (α = 0°) has a Gaussian-shaped profile (Fig. 2A and fig. S7A). The acceptance angle, defined as the full width at half maximum of the ASF, is ~32°. Ommatidium with a large diameter optical waveguide has a large acceptance angle (fig. S8). In other ommatidia, bending losses result in a small portion of incident light being lost in the curved optical waveguides. Ommatidia show peak responses in their ASFs when the incident angles slightly exceed their orientations along the same azimuthal direction (Fig. 2B and fig. S7B). The ASF of the BCE is determined by counting the responses of all the ommatidia, as shown in Fig. 2C, covering a range of 165° × 360° (polar angle × azimuthal angle). The measured result, shown in Fig. 2D, agrees with the calculated ASF. It is worth noting that the effective field of view of every ommatidium is not only determined by the ASF but also related to the acceptable contrast of the image along the orientation. The results indicate that the 3D nature of the BCE camera enables detection across an extremely wide angle of view.

In a natural compound eye, ommatidia are separated by pigment cells and function as independent few-pixel photodetectors. The BCE mimics the anatomical structure of the natural compound eye. The ommatidia are isolated within a black substrate. Cross-talk between the ommatidia in the BCE camera was investigated. Figure 2E schematically shows the experimental setup. A multimode fiber tip connected to a light source was placed in contact with the microlens of an ommatidium, and the resulting light distribution was measured by the camera. The extinction ratio between the ommatidium under illumination and the surrounding ommatidia was higher than 22.1 dB, confirming that the optical cross-talk between the ommatidia was efficiently eliminated (Fig. 2, F and G). The demonstration of light propagation in a U-turn optical waveguide is shown in fig. S9. Each ommatidium in the BCE camera functions as an independent imager. The image collected by the ommatidium is transmitted directly to the image sensor for detection, using the pixels covered by the proximal end of the ommatidium. This setup prevents ghost images from other ommatidia.

The imaging mechanism of the BCE camera was also studied. Simulation models were established for two ommatidia at orientations (α = 0°, β = 0°) and (α = 68.73°, β = 180°). A mask featuring the handwritten digit “6” with a height of 3.5 mm was positioned in front of the ommatidia. Light modulated by the mask illuminated the ommatidia. Ray tracing was conducted to demonstrate the propagation of light within the ommatidia (Fig. 2H). The images collected by the ommatidia in the experiments closely match the simulation results. If no internal reflection occurs, as shown in the central ommatidium (α = 0°, β = 0°), then the rays cross the optic axis after converging, forming a real, inverted, and diminished image at the proximal end of the waveguide. If reflection occurs, then the image is further reversed. Because of the large diameter of the waveguides, there is minimal internal reflection, mitigating optical loss and image distortion. The image distortion resulting from the curved reflecting surface is inevitable but is expected to be corrected during visual cognition. Moreover, the reversed patterns could be correctly restored in the image processing, as demonstrated in fig. S10. The ambiguity in distinguishing similar patterns, e.g., digits “6” and “9,” can be avoided. In practice, undulation across the surface is inevitable in 3D printing. It is a great challenge to polish the internal surfaces of the optical waveguides. The roughness causes undesired scattering in the optical waveguides, as shown in fig. S11. The degradation in image quality, primarily affecting edge sharpness, is both tolerable and reversible. Furthermore, edge sharpness can be restored through edge enhancement techniques.

To have a comprehensive understanding of the imaging capability of the BCE camera, the depth of field was investigated. In the ommatidia, images collected by microlenses are transmitted to the image sensor via optical waveguides; therefore, the length of the optical waveguides determines the distance to the image plane. The optical waveguides connecting the hemispherical surface of the BCE and the image sensor have different lengths ranging from 1.2 to 3 mm. The length of each waveguide is inversely proportional to the distance between the positions of the ommatidia and the central ommatidium. Figure S12 demonstrates the images of a triangular pattern that is placed at different distances to the ommatidia with the orientations of (α = 0°, β = 0°) and (α = 68.7°, β = 0°). The line width of the triangular pattern is 40 μm. The patterns can be clearly recognized by these two ommatidia over object distances ranging from 18.9 to 42.5 mm at (α = 0°, β = 0°) and from 19.9 to 32.5 mm at (α = 68.7°, β = 0°), respectively. The size and line width of the triangles in the image decreases with an increase in object distance. Additionally, the patterns partially appear in the circumjacent ommatidia, providing hints for 3D positioning. The demonstration suggests that the BCE camera is capable of collecting a wealth of information with a wide angle of view and a large depth of field.

### Multistage multi-task deep learning

**Fig. 3.**
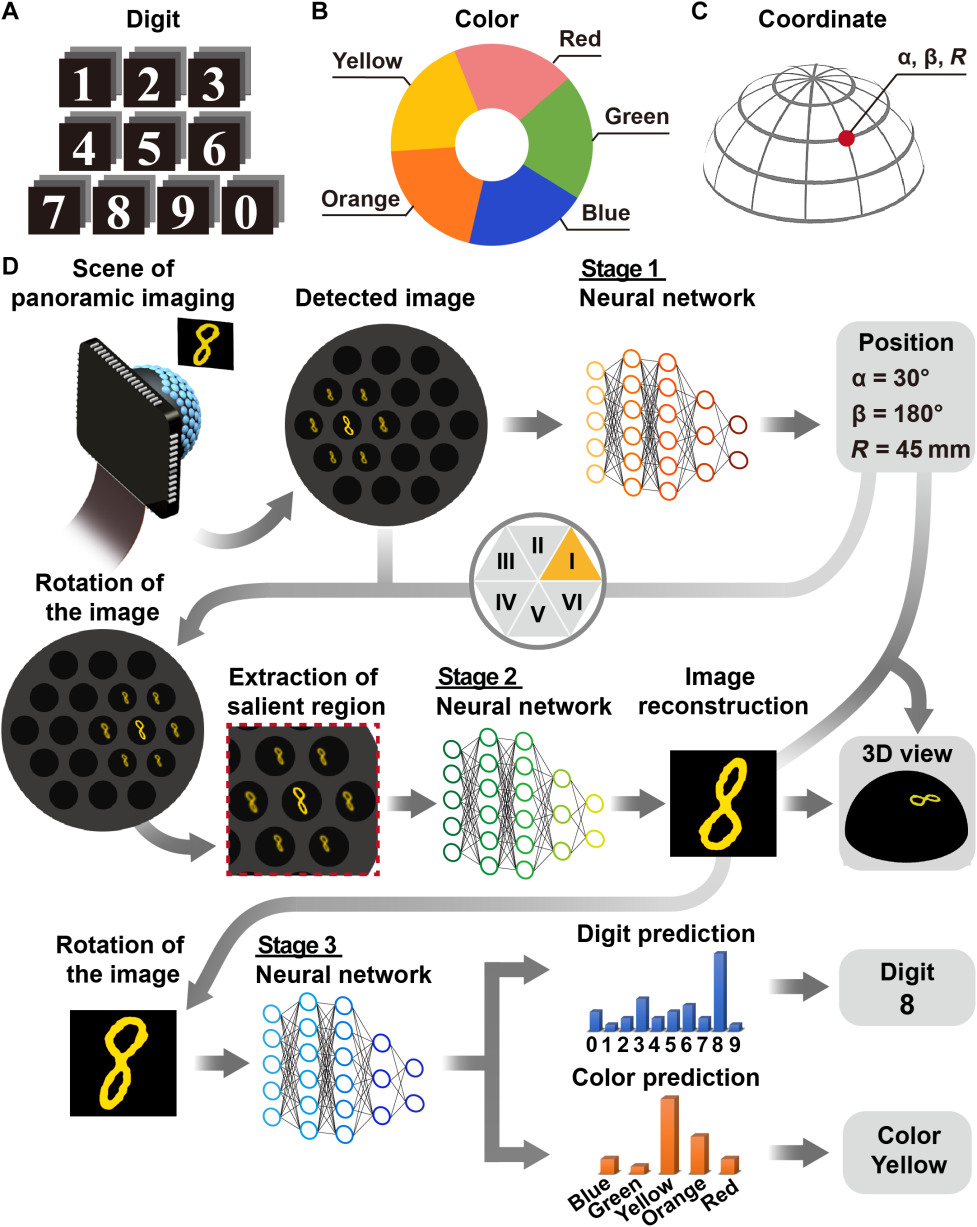
The multistage multi-task visual processing. (**A**) The handwritten digits used for the masks. (**B**) The five colors of the light source. (**C**) The 3D coordinate system. (**D**) The procedure of the deep learning based 3D positioning, image reconstruction, color classification, and pattern recognition.

Visual processing combines the prior knowledge with the visual input to perceive, analyze, and interpret visual patterns, creating meaningful representations. In artificial visual systems, visual processing can be realized by machine learning models that mimic the function and structure of biological neural networks. A machine learning model based on a three-stage deep learning neural network was developed for the BCE camera to perform representative tasks, including 3D position estimation, image reconstruction, pattern recognition, and color classification. The visual system is designed to interpret the visual input from the BCE camera into useful information, i.e., numerical digit, color, and 3D coordinates, and to provide visual representations, i.e., 2D image of the targets and 3D panoramic view (Fig. 3, A to C).

The model is divided into three stages (Fig. 3D), which mirror the functions of the brain. Details of the multistage neural network and the training procedure are provided in Materials and Methods and fig. S13. The first stage, imitating visual attention, directs the visual system to identify and locate critical targets in the wide-view image obtained by the BCE camera. The neural network used for pattern awareness and location prediction is based on the YOLOv5s model (*32*–*34*). Pixel coordinates and the size of the target are calculated to correspond with the orientation, α and β, and the distance to the BCE camera, *R*, respectively; thus, the position of the target in the 3D space is pinpointed. The second stage reconstructs the shape of the target, providing a 2D representation of the visual field. Because the BCE camera with hexagonally arranged ommatidia has rotational symmetry of order 6, the image can be divided into six sections. The image reconstruction for the six sections shares the same operation process. Thus, mastering the image reconstruction for one of the sections, which can significantly improve the learning efficiency of the neural network, is adaptable to the image processing of the entire image. The image obtained by the BCE camera is rotated by a specific angle to position the target into zone I. Subsequently, a sub-image containing the target is extracted from the rotated image. The image extraction allows the neural network to focus on the salient region, efficiently using the computational power on a targeted area of interest in the decomposed image with low redundancy. The extracted sub-image is fed into a residual neural network, for image reconstruction. Moreover, a panoramic image can be generated by mapping the reconstructed images into 3D space. The final stage conducts an abstract analysis of the visual scene. The reconstructed images are used to extract useful information after reverse rotation. A lightweight network in MobileNetV2 architecture was adopted for pattern classification and recognition. The pattern and color of the target are distinguished on the basis of the probability scores predicted by the network.

A large collection of masks of handwritten patterns (MNIST dataset) was prepared for training and testing (fig. S14). The size of the patterns was about 3.5 mm by 4 mm and the line width was 200 μm to 1.1 mm. A total of 6600 images for 200 digit patterns in five colors and different brightness were collected by the BCE camera at 32 positions to train the neural network in the first stage for position prediction. Figure S15 shows that 32 positions are sufficient to achieve high accuracy of positioning. A total of 20,000 images for 3000 digit patterns in five colors and different brightness were collected in zone I to train the neural networks in the second and third stages for image reconstruction and digit and color recognition, respectively. The ground-truth images were generated by filling the patterns with the color and brightness of the light directly obtained by the image sensor without the BCE. The learning curves for the neural networks are illustrated in fig. S16. The training loss rapidly converges to low levels. In the demonstration, 600 images featuring a random combination of 100 digit patterns, five colors, and 20 positions were collected, with all digit patterns and 10 positions not previously used in training. Additionally, 200 images of geometry, alphabet, and insect patterns were used in the testing. All the patterns used for testing were never seen in the training.

### Panoramic imaging and artificial visual cognition

**Fig. 4.**
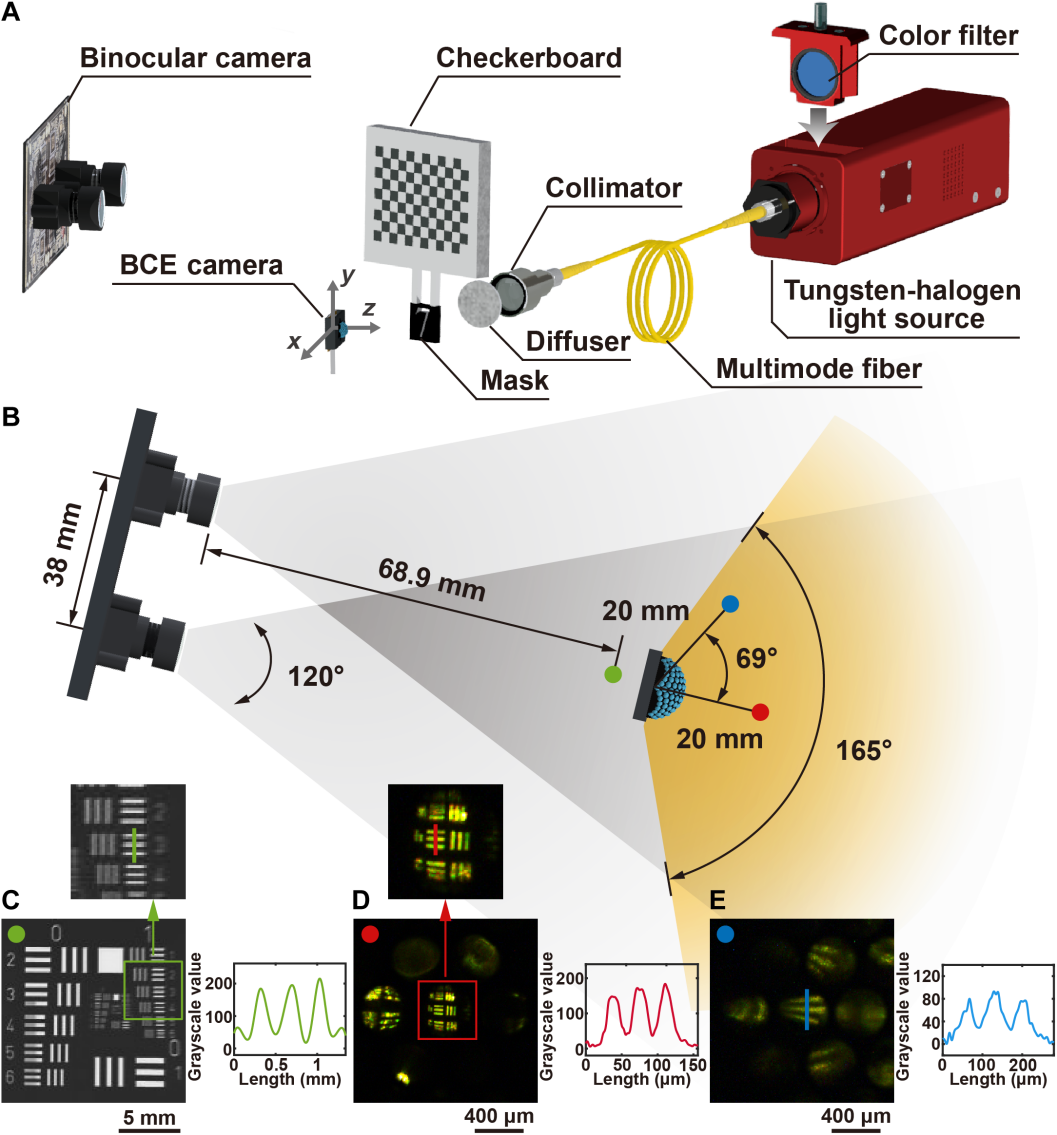
The biomimetic arthropod visual system and binocular imaging system. (**A**) The experimental setup of the imaging system. (**B**) The top view of the imaging systems. (**C**) The measurement of the spatial resolution of the binocular imaging system and the intensity profile along the green line. The resolution target is placed in front of the system [green point in (B)]. (**D** and **E**) The measurement of the spatial resolution of the biomimetic arthropod visual system and the intensity profiles along the red and blue lines. The resolution targets are placed at [α = 0°, β = 0°; red point in (B)] and [α = 69°, β = 180°; blue point in (B)], respectively.

The compact BCE camera has the potential to detect miniature objects with a wide angle of view in a cramped space and capture rich information from the panoramic view. Figure 4A schematically illustrates the imaging setup. The masks were placed at different positions around the BCE camera. Diffused light was used to illuminate the masks. The color of the light was changed by switching the optical filters. A 9 × 12 checkerboard pattern with a size of 27 mm by 36 mm was installed on the top of the masks. A binocular imaging system was used to capture the images of the checkerboard pattern and calculate the 3D positions of the masks (details in Materials and Methods). The calculation results were used as ground-truth data. Figure 4B demonstrates the imaging performances of the BCE camera and the binocular imaging system. The central and peripheral ommatidia of the BCE camera can resolve 22- and 39-μm lines, respectively. The discrepancy in image quality is attributed to the difference in curvature and length between the optical waveguides in which image distortion occurs because of internal reflection. Compared to the binocular imaging system, the BCE camera has unique features as follows: (i) wide angle of view of 165°; (ii) high resolution of ~40 μm (Fig. 4, C to E); (iii) long working distance ranging from 16 to 64 mm (fig. S17); (iv) full vision of 3D perception; (v) high angular perception speed of 9.9 × 10^3^ deg/s (details in Materials and Methods); and (vi) small form factor.

**Fig. 5.**
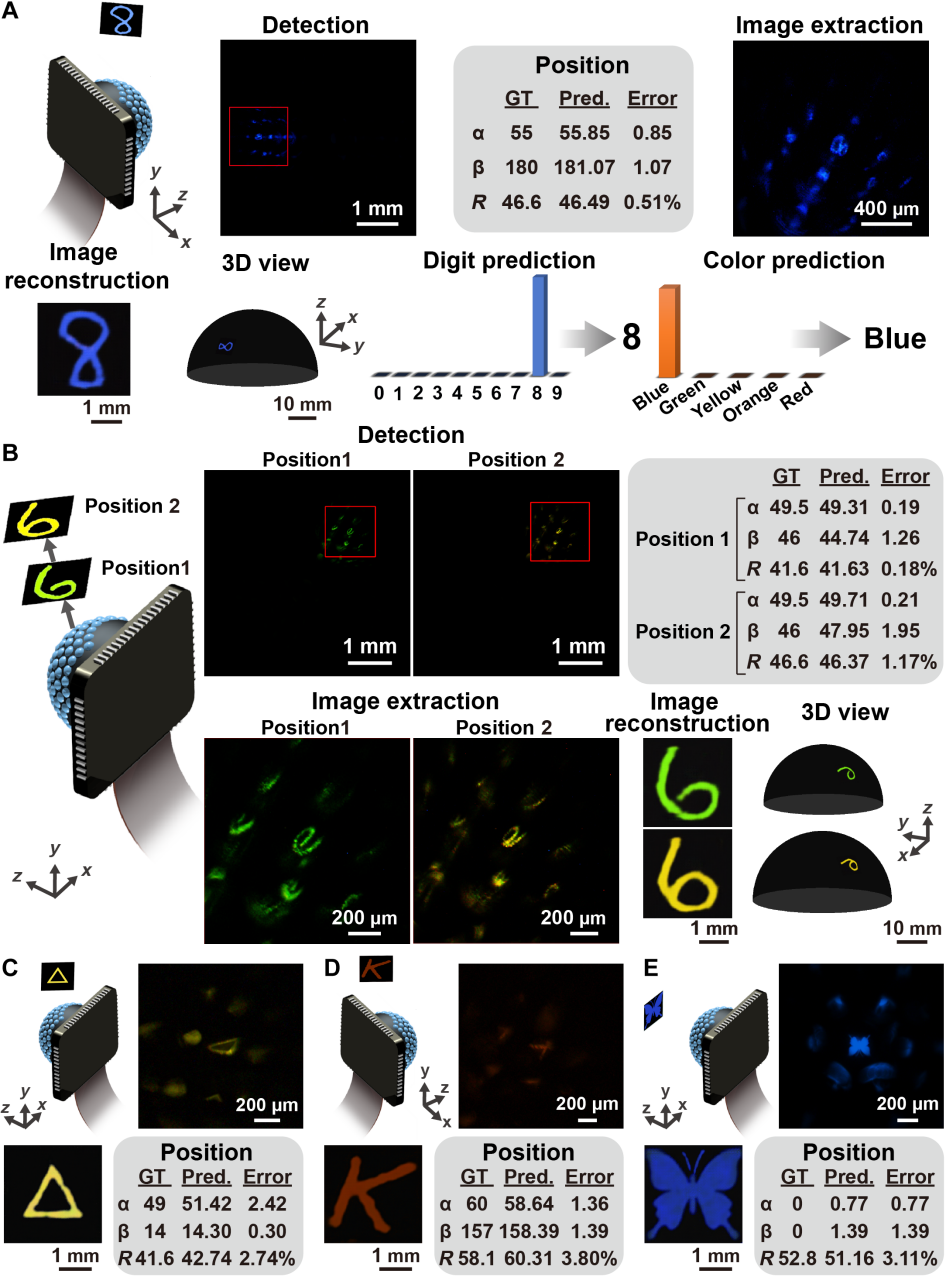
Panoramic imaging and artificial visual cognition using the BCE camera. (**A**) Imaging and recognition of the blue handwritten digit “8” placed on the side of the BCE camera. (**B**) Imaging of the green and yellow handwritten digits “6” placed at different distances to the BCE camera. (**C** to **E**) Detection and reconstruction of the geometry, alphabet, and insect patterns. GT, ground truth; Pred., prediction.

Figure 5 and fig. S18 demonstrate the panoramic imaging and pattern recognition capabilities of the BCE camera. In the detection, an image of 3040 × 3040 × 3 pixels was acquired. Because the neighboring ommatidia had overlapping fields of view, the mask was simultaneously observed by a group of ommatidia. The mask’s pattern appeared repeatedly within the image. Placing the mask in front of the BCE camera (α = 0°, β = 0°) resulted in a symmetrical pattern distribution, due to the axis-symmetrical structure of the BCE, as shown in fig. S18. The symmetry of the pattern distribution decreased as the mask’s orientation moved away from the center, as shown in Fig. 5 (A and B), due to the nonuniform angular sensitivity of the ommatidia. The first-stage image processing involves predicting the 3D position of the mask and extracting a salient region containing the pattern of interest. The location of the region and the pattern distribution in the image hint at the orientation of the mask. In addition, when the mask is positioned far from the BCE camera, more ommatidia can sense the light. Thus, the size of the region is proportional to the distance of the mask from the BCE camera, as shown in Fig. 5B. The 3D position of the mask was calculated. The prediction errors of the orientation, α and β, and the distance, *R*, are less than 2.6° and 2.3%, respectively. Errors for position prediction are shown in fig. S19.

The pattern of the handwritten digits was visible in the image obtained by the BCE camera. The pattern in the image formed by the central ommatidia was clear, while those in the images formed by the peripheral ommatidia were distorted owing to the internal reflection in the curved optical waveguides. The image was rotated to shift the salient region to zone I. Subsequently, a sub-image was extracted from the salient region and processed for image reconstruction and pattern recognition. In stage 2, the image with the size of 256 × 256 × 3 pixels was predicted and righted by the inverse rotation. Details of the pattern could be clearly observed. The distortion of the pattern was efficiently eliminated. The output images closely matched the ground-truth images in terms of shape, color, and brightness. The similarity of the image pairs was quantitatively evaluated (details in Materials and Methods and fig. S20). Structural similarity and 2D correlation are higher than 0.82 and 0.85, respectively. Color similarity was higher than 91.4%. A 3D panoramic image was then generated by projecting the predicted image on a hemispherical dome. The predicted orientation and the distance of the mask to the BCE camera determined the position of the image on the dome and the size of the dome.

The multi-task pattern recognition was conducted in stage 3 using the neural network. Thanks to the realistic reconstruction of the images, the digit and the color of the pattern could be straightforwardly figured out based on probability scoring. Figure S21 and table S1 show the statistics on pattern recognition and color classification. The accuracy of the pattern recognition and color prediction was 95 and 100%, respectively. The central vision (α < 30°) exhibits higher recognition accuracy (≥100%) than the peripheral vision (α > 65°) does (accuracy ≥ 95%) because the image quality obtained in the detection and image reconstruction for the central ommatidia is superior. Figure S22 demonstrates the detection and recognition of multicolor patterns. It is feasible for the biomimetic visual system to distinguish the pattern and calculate the position in 3D space. The image can be clearly reconstructed, and the pattern can be correctly labeled. The colors of the pattern rank top two in the probability scores, indicating that the system has the potential to realize multicolor classification. The computation time used for position prediction, image reconstruction and pattern recognition is 8, 77, and 5 ms per image, respectively.

The imaging performance of the visual system was evaluated under varying illumination conditions, including different degrees of light diffusion and intensity levels. Figure S23 presents images obtained under varying light diffusion. The results indicate that the performance of the visual system is largely insensitive to light diffusion, as the digit patterns were clearly reconstructed. When the light intensity was reduced, the reconstructed images appeared less bright, as shown in fig. S24. Despite the reduced brightness, the digit patterns remained recognizable, and the color was accurately classified across different illumination conditions. Additionally, the system was tested with non-digit patterns that were not included in the training dataset. Figure 5 (C to E) and fig. S25 show the reconstructed images of these patterns, demonstrating that the visual system could achieve high fidelity in reconstruction, even without prior exposure to these designs. However, pattern recognition was not performed, as the system had not been trained on these specific patterns. The quality of the reconstructed images was assessed by comparing them to the ground-truth images. The visual system achieved high structural similarity (>0.86), 2D correlation (>0.89), and color similarity (>95.2%). These results highlight the system’s robustness in detecting and reconstructing various patterns under diverse illumination conditions.

**Fig. 6.**
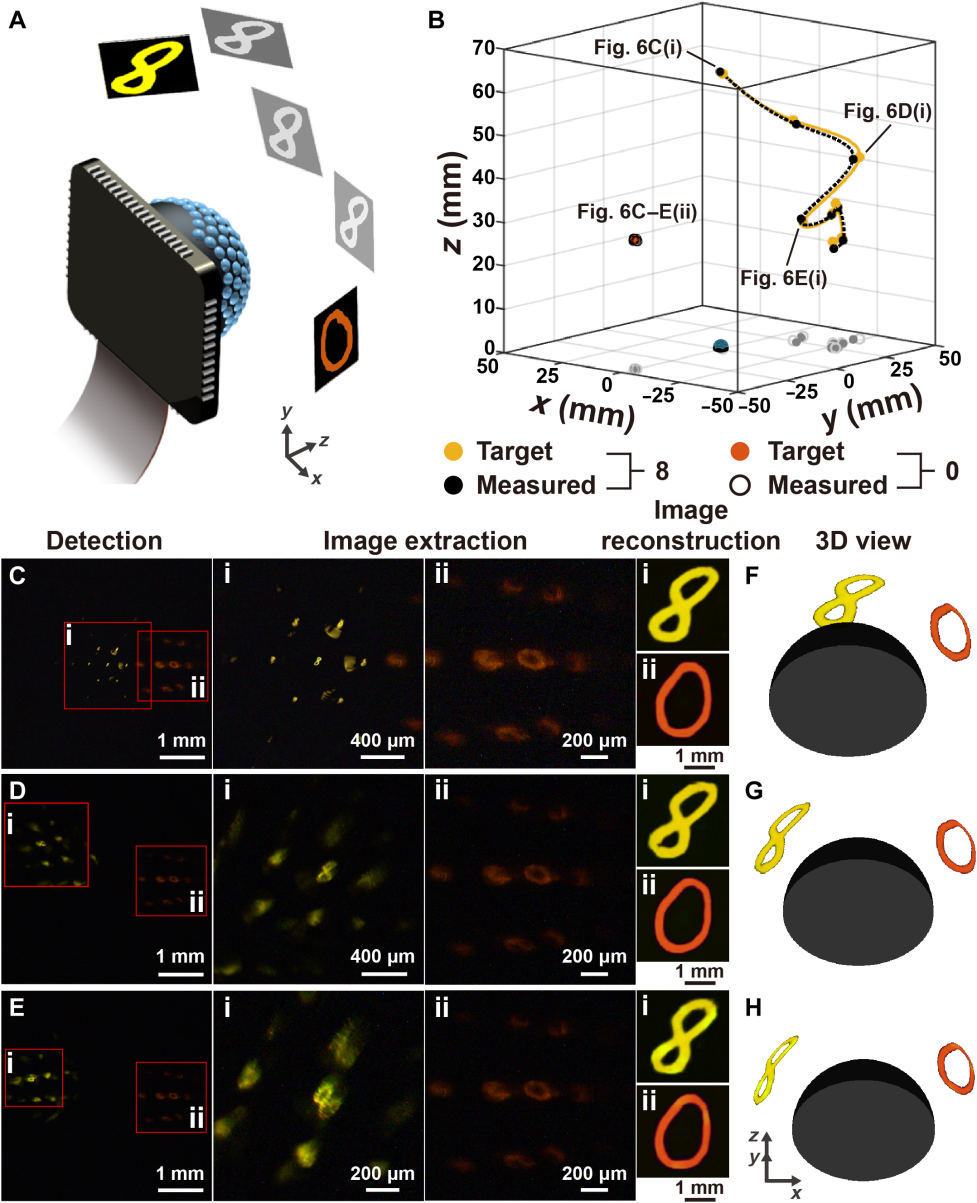
Tracking the two digits using the BCE camera. (**A**) Schematic diagram of the tracking experiments. The mask with the orange digit “0” was fixed, while the mask with the yellow digit “8” moved away from the center. (**B**) The spatial positions of the two digits and the movement path of the digit “8.” The yellow and black solid dots are the target and predicted positions for the digit “8,” respectively. The orange solid dots and the black circles are the target and predicted positions for the digit “0,” respectively. (**C** to **E**) The images captured by the BCE camera and the reconstructed digit patterns, (i) digit “8” and (ii) digit “0,” at different moments. (**F** to **H**) 3D views of mask movement reconstructed by the visual system. The orientation is truly presented while the scale is arbitrary. The 3D view with true scale is shown in fig. S26.

Furthermore, the BCE camera was used in a challenging task to monitor multiple objects and track their 3D positions. In the demonstration, a mask with the orange digit “0” was fixed on the side of the BCE camera, while another mask with the yellow digit “8” spiraled around the BCE camera along an irregular path, as illustrated in Fig. 6A. The images were collected at different moments. Figure 6B depicts the predicted positions of both masks and the movement path. The good agreement with the ground-truth data verifies that the BCE camera has the capability of multiple object tracking. Even if the two masks were close to each other, the BCE camera could precisely mark the two salient regions as shown in Fig. 6C. The low deviation in the 3D position prediction for the fixed mask substantiates the high robustness of the BCE camera. The images for both digits were separately reconstructed. The two digits are clearly presented (Fig. 6, C to E). The imaging performance is tolerable to the partial overlap of the salient regions. No interference is noticed in the predicted images. A high consistency in shape (structural similarity > 0.87 and 2D correlation > 0.91) and color (color similarity > 96.3%) of the pattern could be guaranteed at different positions, ensuring high-quality imaging over a wide-angle view. The images shown in Fig. 6 (F to H) and fig. S26 exhibit the 3D visual perspective of the BCE camera when observing the two digits.

Because the acceptance angle of the ommatidia is larger than the interommatidial angle (13.74°), the neighboring ommatidia captures the images of the objects placed closely. Artifacts of the undesired pattern appear around the target pattern. The performances of the biomimetic visual system are quantitatively evaluated for 3D positioning and imaging of two objects with the minimum separation angle, as listed in table S2. If objects are located with a separation angle larger than 15°, the visual system can accurately distinguish between them, as demonstrated in figs. S27 and S28. The existence of the artifacts in the extracted salient region has a low influence over image reconstruction. The BCE camera, exhibiting the outstanding capability for visual attention and cognition with high-resolution imaging, high-precision 3D positioning, and high-accuracy pattern recognition, would be especially attractive in robotic platforms ranging from capsule endoscopy to miniature terrestrial and aerial vehicles (*35*–*40*).

## DISCUSSION

In summary, we have developed a biomimetic arthropod visual system designed to emulate the functions of the natural compound eye and brain in arthropods, enabling panoramic imaging, depth perception, and information interpretation. The BCE camera, as a key device in the biomimetic arthropod visual system, is fabricated on the basis of an efficient, cost-effective microfluidic-assisted 3D printing technique and complementary metal oxide semiconductor manufacturing process. The BCE seamlessly integrates with a single planar image sensor, eliminating the need for bulky relay lenses or complex 3D photodetectors. Instead of compromising ommatidium size for quantity, microlenses and optical waveguides with large apertures were designed in the BCE for low insertion loss and a large number of effective pixels. The improvements in structural design empower the BCE to have a high spatial resolution for imaging, retaining the feature of large angle of view. In contrast to the advanced insect-inspired ultrathin cameras using planar microlens arrays (*8*, *41*, *42*), the BCE camera exhibits comparable image quality with larger view angles, but the BCE camera is thick due to the hemispherical profile of the compound eye. The BCE can be optimized in a different shape to reduce the height and completely cover the sensing area of the image sensor, making full use of the imaging pixels. For example, a thin hemi-ellipsoidal BCE would be suitable to match a rectangular image sensor.

The BCE camera has demonstrated the extraordinary capability of full-color, wide-angle imaging with high resolving power and a large number of pixels. A wealth of information can be acquired by using the BCE camera. The discrepancy in image quality among the ommatidia is attributed to the difference in image transmission paths inside the BCE. An optimized structural design of the ommatidia with optical waveguides of uniform length and straight shape might be a desired solution. Moreover, artificial intelligence was applied to boost the capability of the BCE camera in all aspects of visual processing. A multistage neural network–based algorithm has been developed for targeting regions of interest, reconstructing high-quality images, tracking 3D motion and recalling visual representations. Table S3 lists existing state-of-the-art BCE-based visual systems for a comparison of imaging capability and function demonstration.

It is feasible to simultaneously locate multiple targets in the 3D space and independently reconstruct the image of each target without interference, ensuring that useful information is accurately interpreted. The visual attention allows comprehensive analysis of the specific targets, but the scene around the targets is neglected. The BCE camera as a multi-ocular system has stereo vision and has the capability to establish a model of the 3D scene with a 360° view. To accomplish this complicated task, unsupervised learning and self-supervised learning would be suitable approaches that could efficiently eliminate heavy workloads in ground-truth dataset acquisition, feature extraction, and target labeling. Despite the compactness of the BCE camera, it would be a challenging step for the miniaturization of the entire visual system, particularly for artificial intelligence–based signal processing. The neural networks can be optimized to save computing resources and reduce computation time by pruning less important weights and reducing the dimensionality of the image features (*43*, *44*). The low latency of image processing could allow the BCE camera to operate in real time. Future advancements could involve integrating an optical neural network and incorporating a photoelectronic chip into the BCE camera, offering promising solutions for enhancing miniaturization and performance (*45*–*48*). We anticipate that ongoing developments in biomimetic arthropod visual systems, such as the BCE camera, will drive profound innovations in fields such as surveillance, robotic navigation, and medical diagnostics.

## MATERIALS AND METHODS

### Design and fabrication of the BCE camera

Figure 1D shows the structure of the BCE camera. Figure S1 illustrates the fabrication process for the BCE camera, as follows: (i) A mold with an open hemispherical pit and 127 concave microholes arranged omnidirectionally on the bottom of the pit was designed by Autodesk Inventor and 3D printed by a projection micro-stereolithography 3D printer (nanoArch P140, BMF Precision Technology Co., China). There were six slots on the surface of the mold. (ii) Photoresist (SU8 2005, Microchem, USA) was added to the pit of the mold. (iii) The mold was spun at 6000 rpm for 40 s. (iv) After 30 min of stabilization in the dark, the mold was exposed to ultraviolet (UV) light for 10 min to cure the photoresist. (v) A hemispherical substrate consisting of 127 hollow pipelines was designed and 3D printed using a UV curable diacrylate polymer (refractive index *n*_Substrate_ = 1.46). There were six auxiliary supports around the substrate (Fig. 1E). Then, the hemispherical substrate was placed into the pit of the mold. The alignment of the concave microholes and the pipelines was realized by placing the auxiliary supports in the slots on the mold. The precision of the alignment could be guaranteed, as shown in fig. S2. (vi) The mold containing the substrate was immersed into liquid-state silicone (Gelest OE 50, Gelest Inc., USA) and evacuated at −0.1 MPa for 20 min to ensure that the pipelines and the microholes were filled with the silicone. The silicone was prepared by mixing the vinyldimethylsiloxane terminated phenyl(-chlorophenyl)siloxane-dimethylsiloxane copolymer and hydride terminated methylhydrosiloxane-phenylmethylsiloxane copolymer with a weight ratio of 1:1. The refractive index of the silicone is *n*_Silicone_ = 1.50. (vii) The silicone was cured at 55°C for 4 hours. (viii) The BCE was separated from the mold. (ix) A frame for installing the BCE and the image sensor (IMX577, Sony, Japan) was designed and 3D printed. The cover of the frame has six slots for mounting the BCE. Black adhesive (K-704, Kafuter, Guangdong Hengda New Materials Technology Co. Ltd., China) was used to seal the frame and the BCE. (x) The image sensor was placed in the base of the frame. The frames containing the BCE and the image sensor were assembled, forming a BCE camera.

The ommatidia are hexagonally arranged in the BCE. The layer number, *m*, of the concentric hexagons is determined by the size of the BCE, *D*, the diameter of the proximal end of the optical waveguide, *d*_B_, and the separation between the proximal ends of the waveguides, *d*_S_, and can be expressed as

$$m=\left\lfloor \frac{D-d_{B}}{2\left(d_{B}+d_{S}\right)}\right\rfloor$$ (1)

where ⌊·⌋ is the greatest integer function. Thus, the number of the ommatidia for the BCE can be calculated as

$$M=3m^{2}+3m+1$$ (2)

In the design of the microlenses, the relation between focal length, *f*, and image distance, *v*, is suggested as *f* < *v* < 2 *f*. The minimum and maximum image distance is determined by the minimum and maximum of the optical waveguides, *L*_min_ and *L*_max_, plus the thickness of the protection glass on the image sensors, *T*_Glass_. Therefore, the range of the focal length is (*L*_max_ + *T*_Glass_)/2 < *f* < *L*_min_ + *T*_Glass_. The curvature radius of the microlenses can be designed as *r* = *f*(*n*_Silicone_ − 1)/*n*_Silicone_. The maximum angular perception speed is defined as ω_max_ = *R*_Sensor_*AOV*, where *R*_Sensor_ is the response of the sensor (*49*).

### Experimental setup of the imaging system

The schematic diagram of the imaging system is illustrated in Fig. 4A. Two tungsten-halogen light sources (SLS201L/M, Thorlabs Inc., USA) were used to generate broadband white light. Color glasses were used in the light source to change the color of the light to blue, green, yellow, orange, and red, respectively. The light was output from collimators (F810FC-543, Thorlabs Inc., USA) via multimode fibers (M74L01, Thorlabs Inc., USA). After passing through an optical diffuser, the light illuminated the mask. The mask was placed around the BCE camera. There was a 9 × 12 checkerboard pattern on the top of the mask. The size of the black and white squares on the checkerboard pattern was 3 mm. A binocular imaging system was installed behind the BCE camera. In the binocular imaging system, the distance between the two cameras was 38 mm. The focal length of the lenses was 2.3 mm. The pixel size of the image sensors used in the binocular imaging system was 1.55 μm by 1.55 μm. The depth resolution with the change of object distance is plotted in fig. S29. The USAF1951 resolution target (Thorlabs Inc., USA) was used to evaluate the spatial resolution of the biomimetic arthropod visual system and binocular imaging system. The binocular imaging system had sufficient spatial resolution to provide accurate 3D positions as ground truth (fig. S30). A customized MATLAB program was developed to measure the positions of the checkerboard pattern and the mask in real time.

### Image processing

Figure 3 and fig. S13 schematically illustrate the block diagram of the image processing for 3D positioning, image reconstruction, color classification, and pattern recognition. In the demonstration, a laptop with central processing unit of Intel Core i7-1165G7 @ 2.80GHz, random-access memory of 32 GB, and graphics processing unit of NVIDIA GeForce MX450 was used for processing. The color images obtained by the BCE camera are truncated to the size of 3040 × 3040 × 3 pixels. YOLOv5s model is applied to figure out the location of the target in the image. In the YOLOv5s model, the image is resized to 512 × 512-pixel grayscale image for processing, and the feature maps with the size of 32 × 32 × 1024 are generated. The features are aggregated by up-sampling and down-sampling process. Then, three feature maps in different scales containing the central coordinate (*x*_c_, *y*_c_) and the size (*w* × *w*) of the target and the confidence of the prediction are generated. A sigmoid activation function is followed to quantitatively calculate the probability of the predicted location and size, forming a bounding box for the prediction with the highest probability of highlighting the target.

The coordinate and the size of the target in the 2D image calculated by the YOLOv5s model are converted to 3D polar coordinates (α, β, *R*) by the following formulas

$$\alpha =\text{arcsin}\left(\frac{\sqrt{{x_{c}}^{2}+{y_{c}}^{2}}}{R}\right)$$ (3)

β={arctan(ycxc),xc>0180°+arctan(ycxc),xc<090°,xc=0 and yc>0270°,xc=0 and yc<0 (4)

R=pw (5)

where *p* is a scale factor for the conversion between the distance and the number of pixels, corresponding to the depth resolution. The value is manually given in the training. There is a compromise between the depth resolution and the range of the distance, i.e., *R*_max_ = *p*(*n*_Det_ − *n*_OmmDia_)/2, where *n*_Det_ is the pixel dimension of the detected image in one dimension and *n*_OmmDia_ is the pixel number of the ommatidia diameter in the detected image.

Then, the image is rotated to shift the target to zone I. The rotation angle can be calculated as

$$\theta =-\left\lfloor \frac{\beta }{60{}^{\circ}}\right\rfloor \cdot 60{}^{\circ}$$ (6)

where ⌊·⌋ is the floor function to output the greatest integer less or equal to the input. After the image rotation, the coordinate of the target (*x′*, *y′*) becomes

$$\left(\begin{array}{c} x\prime \\ y\prime \end{array}\right)=\left(\begin{array}{cc} \text{cos}\;\theta & -\text{sin}\;\theta \\ \text{sin}\;\theta & \text{cos}\;\theta \end{array}\right)\left(\begin{array}{c} x\\ y \end{array}\right)$$ (7)

The region of interest containing the target is extracted from the image with the size of (*w* × *w*). The sub-image is resized to 1024 × 1024 × 3 pixels and fed into the residual neural network for image reconstruction. Figure S13B shows the architecture of the residual neural network model. The backbone is based on ResNet34 for feature extraction (*50*). Up-sampling layers and residual blocks are followed to predict an image with a size of 256 × 256 × 3.

After that, the output image is reversely rotated to the initial state. A panoramic view can be generated by establishing a hemisphere and 2D-to-3D mapping the reconstructed image onto the hemisphere using Eqs. 3 to 5. The panoramic view has the pixel number of $\left\lceil 2,\pi ,{\left(n_{\text{Rec}}R/L\right)}^{2}\right\rceil$, where ⌈·⌉ is the ceiling function to output the least integer greater or equal to the input, *n*_Rec_ is the pixel dimension of the reconstructed image, i.e., *n*_Rec_ = 256 pixels, and *L* is the side length of the mask.

Subsequently, the 2D image is input into a probabilistic neural network, MobileNetV2, for pattern recognition (*51*, *52*). The MobileNetV2 model consists of a convolution layer and 19 residual bottleneck layers. At the end of the neural network, two blocks containing average-pooling, dropout, and linear-regression functions are used to provide probability scores for 10-digit and five-color classification. The digit and color are determined on the basis of the highest probability scores.

### Evaluation metrics

The prediction errors of polar angle (α), azimuthal angle (β), and distance *R* are calculated as

$$\varepsilon_{\alpha }=|\alpha -\alpha_{\text{GT}}|$$ (8)

$$\varepsilon_{\beta }=|\beta -\beta_{\text{GT}}|$$ (9)

$$\varepsilon_{R}=\frac{|R-R_{\text{GT}}|}{R_{\text{GT}}}\times 100\%$$ (10)

where α_GT_, β_GT_, and *R*_GT_ are the ground truth of the polar angle, azimuthal angle, and distance obtained by the binocular imaging system.

The reconstructed image, *T*, is compared with the ground-truth image, *O*, which is generated by filling in the masks with the color of the incident light. The structural similarity (SSIM) index is defined as

SSIM(T,O)=l(T,O)c(T,O)s(T,O) (11)

where

$$l\left(T,O\right)=\frac{2\mu_{T}\mu_{O}+C_{1}}{{\mu_{T}}^{2}+{\mu_{O}}^{2}+C_{1}}$$ (12)

$$c\left(T,O\right)=\frac{2\sigma_{T}\sigma_{O}+C_{2}}{{\sigma_{T}}^{2}+{\sigma_{O}}^{2}+C_{2}}$$ (13)

$$s\left(T,O\right)=\frac{\sigma_{\text{TO}}+C_{3}}{\sigma_{T}\sigma_{O}+C_{3}}$$ (14)

where μ*_T_* and μ*_O_* are the mean luminance of the two images; σ*_T_* and σ*_O_* are the SD of the luminance of the two images; σ*_TO_* is the covariance between the luminance of the two images; and *C*_1_, *C*_2_, and *C*_3_ are three positive constants used to avoid a null denominator. The SSIM ranges from 0 to 1. If SSIM = 1, then the two images are identical.

The fidelity of the images is evaluated by calculating the 2D correlation between the reconstructed image and the ground-truth image as follows

$$\text{corr}2D=\frac{\sum_{m=1}^{M}\sum_{n=1}^{N}\left[T\left(m,n\right)-\mu_{T}\right]\left[O\left(m,n\right)-\mu_{O}\right]}{\mathrm{MN}\sigma_{T}\sigma_{O}}$$ (15)

where *M* and *N* are the number of pixels in row and column. If corr2D is 1, the reconstructed image is consistent with the ground-truth image.

The color similarity (CS) index is defined as

$$\begin{array}{c} \text{CS}=\left(1-\frac{\sqrt{\sum_{i=R,G,B}{\left(T_{i}-O_{i}\right)}^{2}}}{\sqrt{\sum_{i=R,G,B}\text{max}{\left(O_{i},255-O_{i}\right)}^{2}}}\right)\times 100\% \end{array}$$ (16)

where (*T*_R_, *T*_G_, *T*_B_) and (*O*_R_, *O*_G_, *O*_B_) are the average values of the red, green, and blue channels for the pattern in the reconstructed image and the ground-truth image, respectively.

The accuracy of the number and color prediction in pattern recognition is calculated as

$$\text{Accuracy}=\frac{N_{\text{Acc}}}{N_{\text{Total}}}\times 100\%$$ (17)

where *N*_Acc_ is the number of accurate predictions and *N*_Total_ is the total number of patterns for testing.

## Supplementary Materials

### This PDF file includes:

Figs. S1 to S30 Tables S1 to S3 References
