# Supplementary material for: Seeing through arthropod eyes: An AI-assisted, biomimetic approach for high-resolution, multi-task imaging
Source: Sci Adv. 2025 May 21;11(21):eadt3505. doi: 10.1126/sciadv.adt3505 (PMC12094235; doi:10.1126/sciadv.adt3505)
Supplement: Supplementary file 1 — Figs. S1 to S30 Tables S1 to S3 References [file sciadv.adt3505_sm.pdf]

Science Advances
AAAS

Supplementary Materials for

Seeing through arthropod eyes: An AI-assisted, biomimetic approach for high-resolution, multi-task imaging

Yan Long *et al.*

Corresponding author: Tony Jun Huang, tony.huang@duke.edu; Dawei Zhang, dwzhang@usst.edu.cn

Sci. Adv. **11**, eadt3505 (2025)
DOI: 10.1126/sciadv.adt3505

This PDF file includes:

Figs. S1 to S30
Tables S1 to S3
References

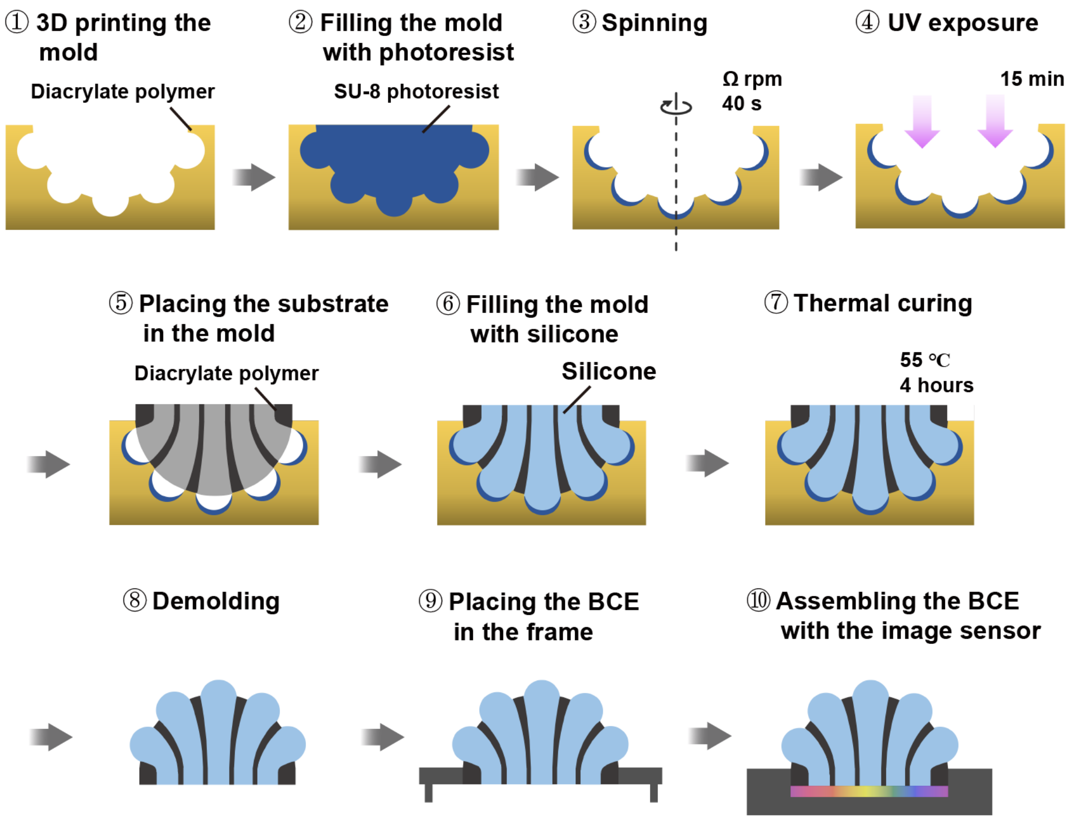

Fig. S1. Fabrication procedure of the BCE-camera. Step 1 - Step 4: formation of the mold for the BCE. Step 5 - Step 8: formation of the BCE. Step 9 and Step 10: installation of the BCE-camera.

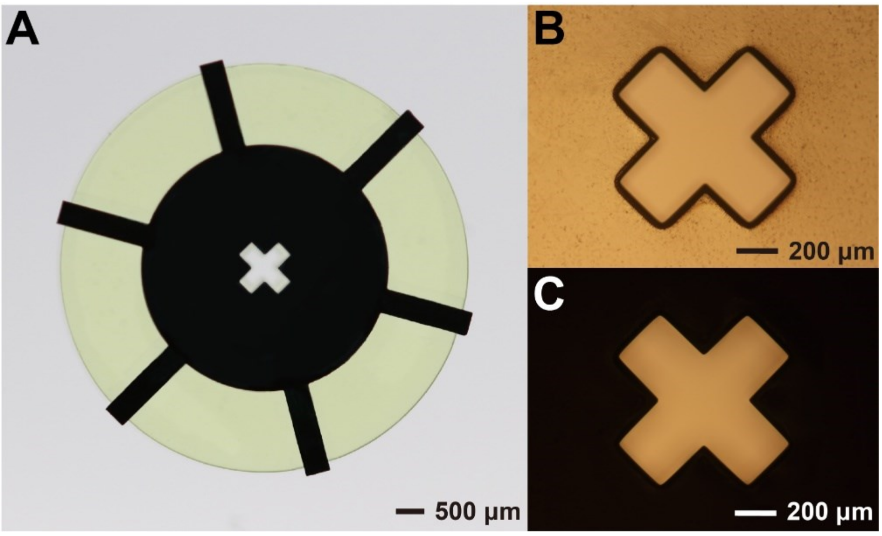

Fig. S2. Demonstration of the alignment of the two test pieces. (A) Photo of the two test pieces stacked together. The test piece with six auxiliary supports was placed on the other test piece with six slots on the surface. **(B)** and **(C)** Microscopic images of the two test pieces before and after stacking. The shape of the cross '×' was unchanged after stacking the test pieces together, indicating that the two pieces were well aligned.

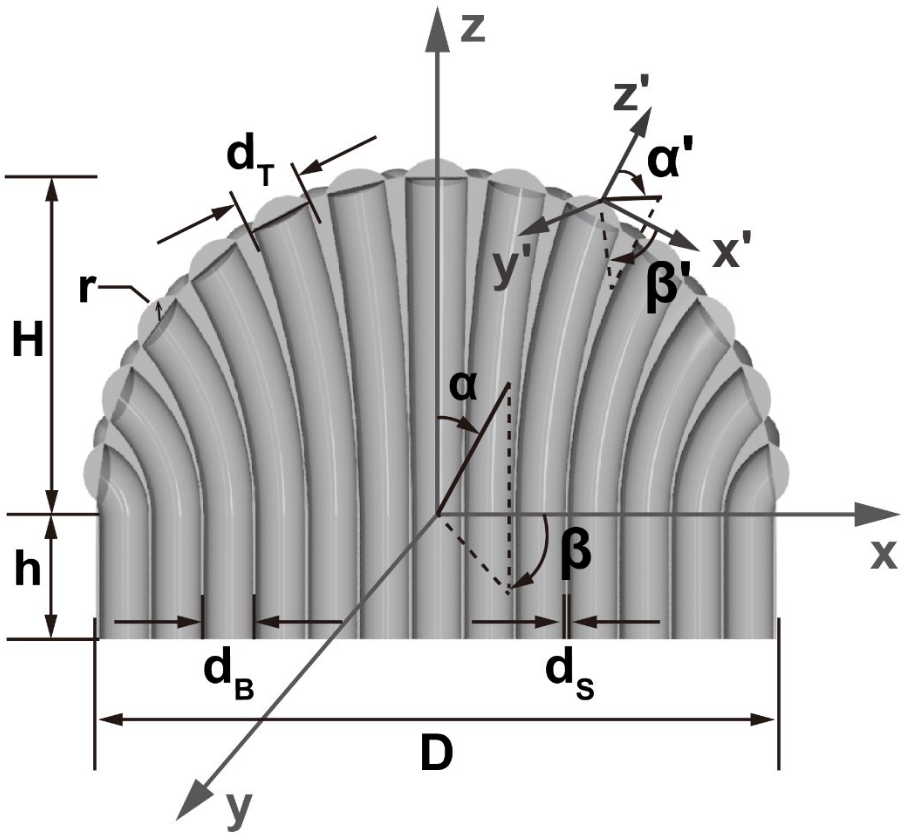

Fig. S3. Cross section of the substrate for the BCE.

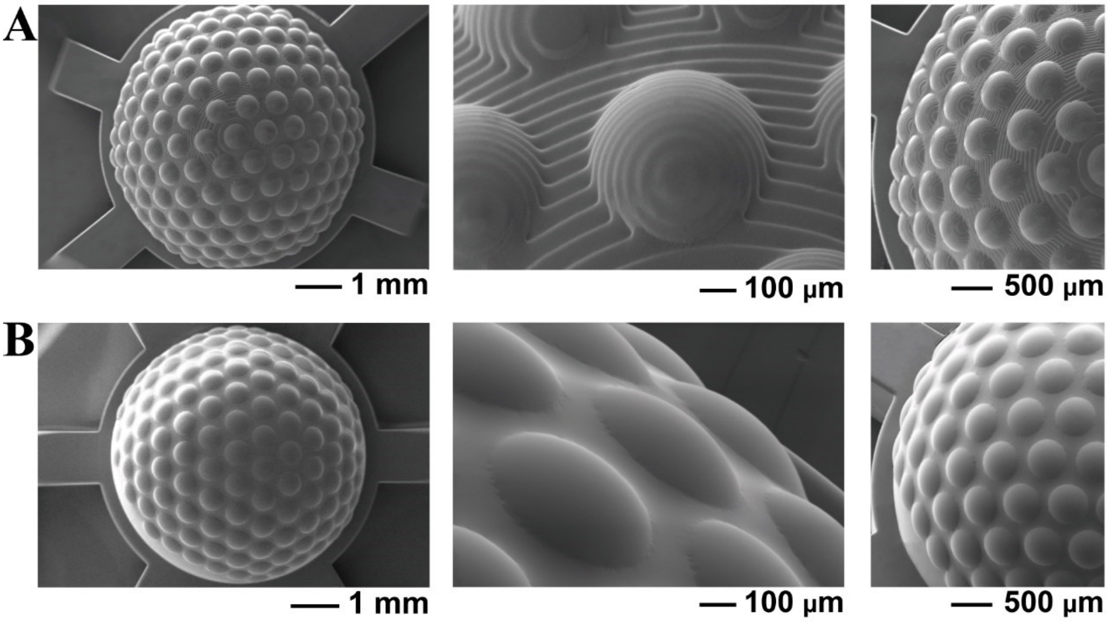

Fig. S4. SEM images of the polydimethylsiloxane (PDMS) demolded from the mold. (A) The PDMS is demolded from the 3D printed mold. The surface undulation is attributed to the 3D printing. **(B)** The PDMS is demolded from the 3D printed mold that is filled with the photoresist and spun to form the concavity of the microlenses. The rightmost image is the same as **Fig. 1F**.

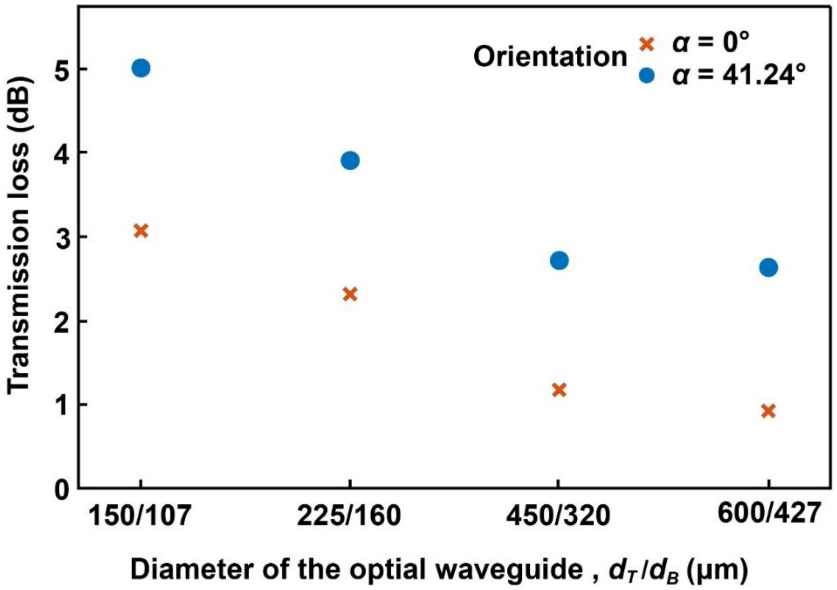

Fig. S5. The optical loss in the ommatidia with the optical waveguides of different diameters.

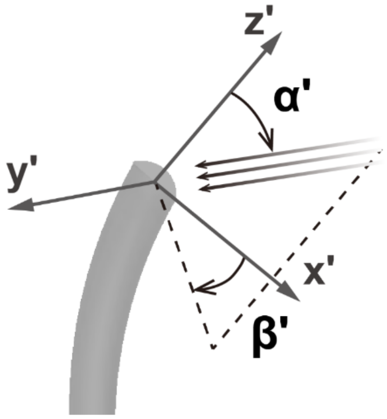

Fig. S6. The ommatidium model for calculating the angular sensitivity function. The collimated light illuminates the ommatidium with different incident angles.

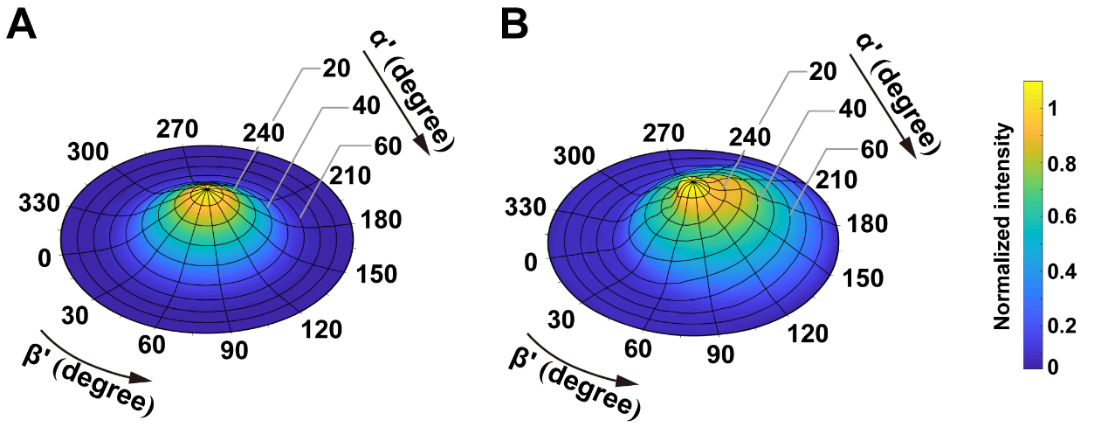

Fig. S7. The angular sensitivity function (ASF) of the ommatidia. (A) The ASF for the central ommatidium (α = 0°, β = 0°) is defined as a Gaussian function, *i.e.*, $ASF_{CentralOmm} = I_{max}\left(e^{-\frac{(x-x_{max})^2}{2\sigma^2}} + e^{-\frac{(y-y_{max})^2}{2\sigma^2}}\right)$, where I_{max} is the peak intensity of the distribution, (x_{max} , y_{max}) is the position of the peak and σ is the width of the Gaussian distribution. **(B)** The ASF for the ommatidium at the orientation of (α = 68.73°, β = 180°). The angular sensitivities for peripheral ommatidia are in irregular profiles. The ASFs for the peripheral ommatidia are fitted using biharmonic spline interpolation.

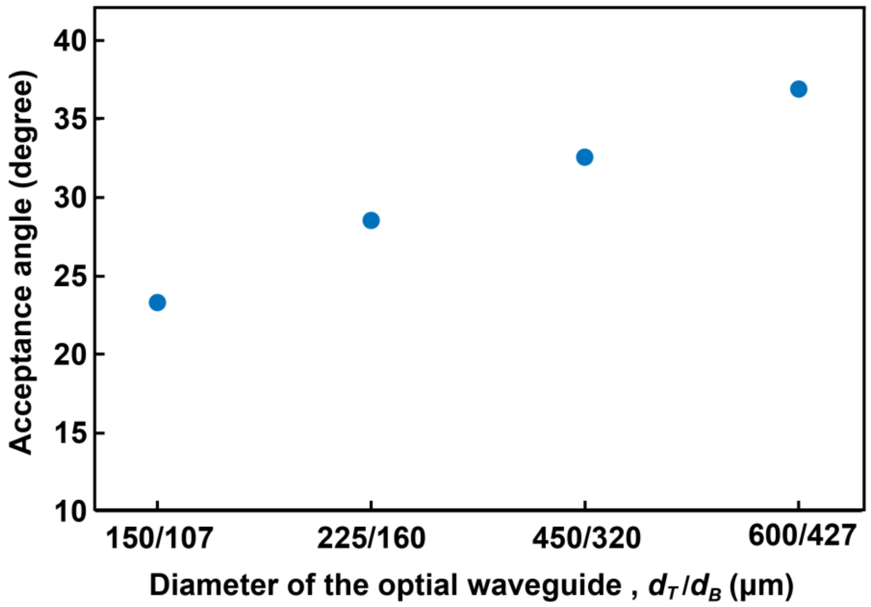

Fig. S8. The influence of the diameter of the optical waveguide in the ommatidia over the acceptance angle.

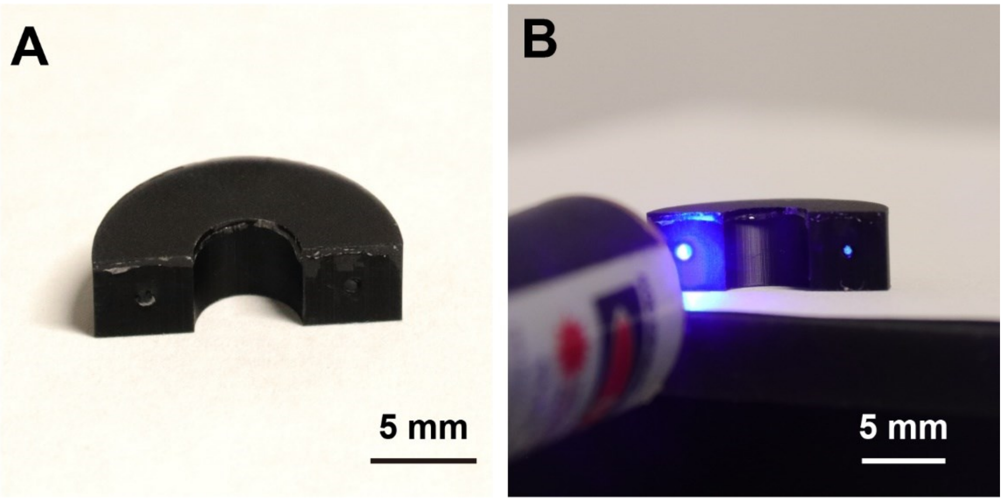

Fig. S9. Demonstration of the light transmission in a U-turn optical waveguide. The diameter of the optical waveguides is 400 μm. The radius of the turn is 5 mm. **(A)** The 3D printed piece has a hollow pipeline filled with silicone, forming a U-turn optical waveguide. **(B)** The light illuminates one end of the optical waveguide and can be observed from the other end.

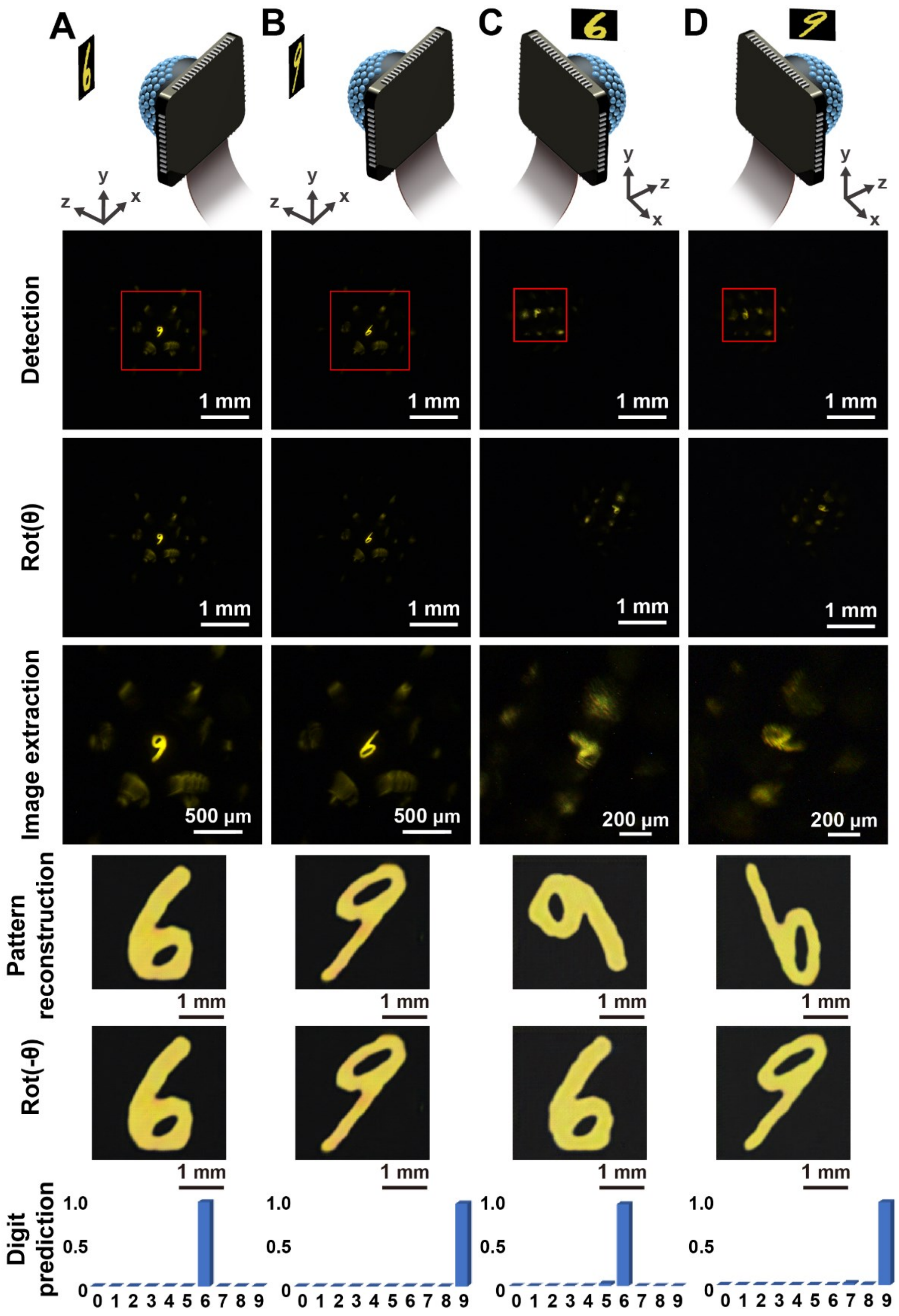

Fig. S10. The entire procedure of the 3D positioning, image reconstruction and digit recognition of digit patterns '6' and '9'. (A) Digit pattern '6' is in front of the BCE-camera with the position of α=0°, β=0° and R=53.8 mm. **(B)** Digit pattern '9' is in front of the BCE-camera with the position of α=0°, β=0° and R=53.8 mm. **(C)** Digit pattern '6' is on the side of the BCE-camera with the position of α=50°, β=166° and R=41.3 mm. **(D)** Digit pattern '9' is on the side of the BCE-camera with the position of α=50°, β=166° and R=41.3 mm.

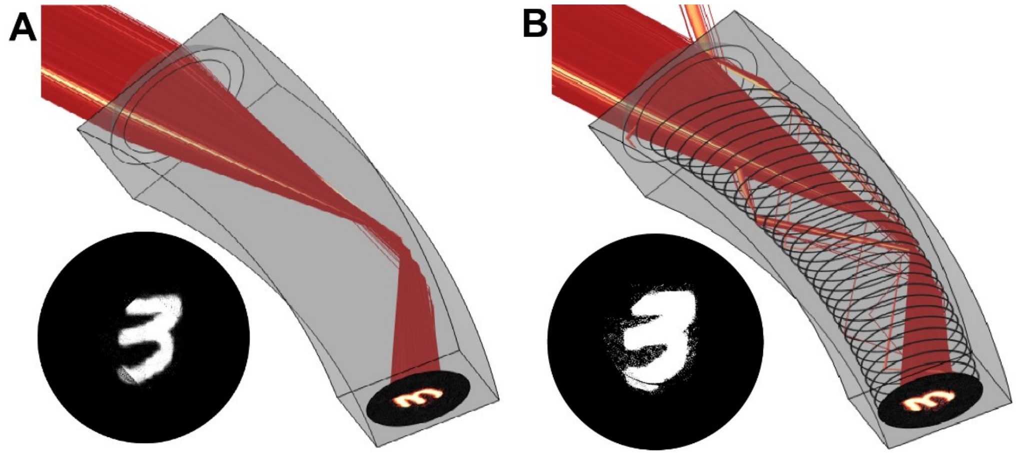

Fig. S11. Simulation of the light propagation in the ommatidia and the images captured at the proximal ends of the optical waveguides with smooth and rough surfaces. The orientation of the ommatidium is α=68.73°. **(A)** The optical waveguide has an ideal smooth surface. **(B)** The optical waveguide has a rough surface. The rough surface has the profile of periodic undulation with a peak-to-valley height of 8.3 μm. The surface roughness was measured by the surface roughness tester (SJ410, Mitutoyo, Japan).

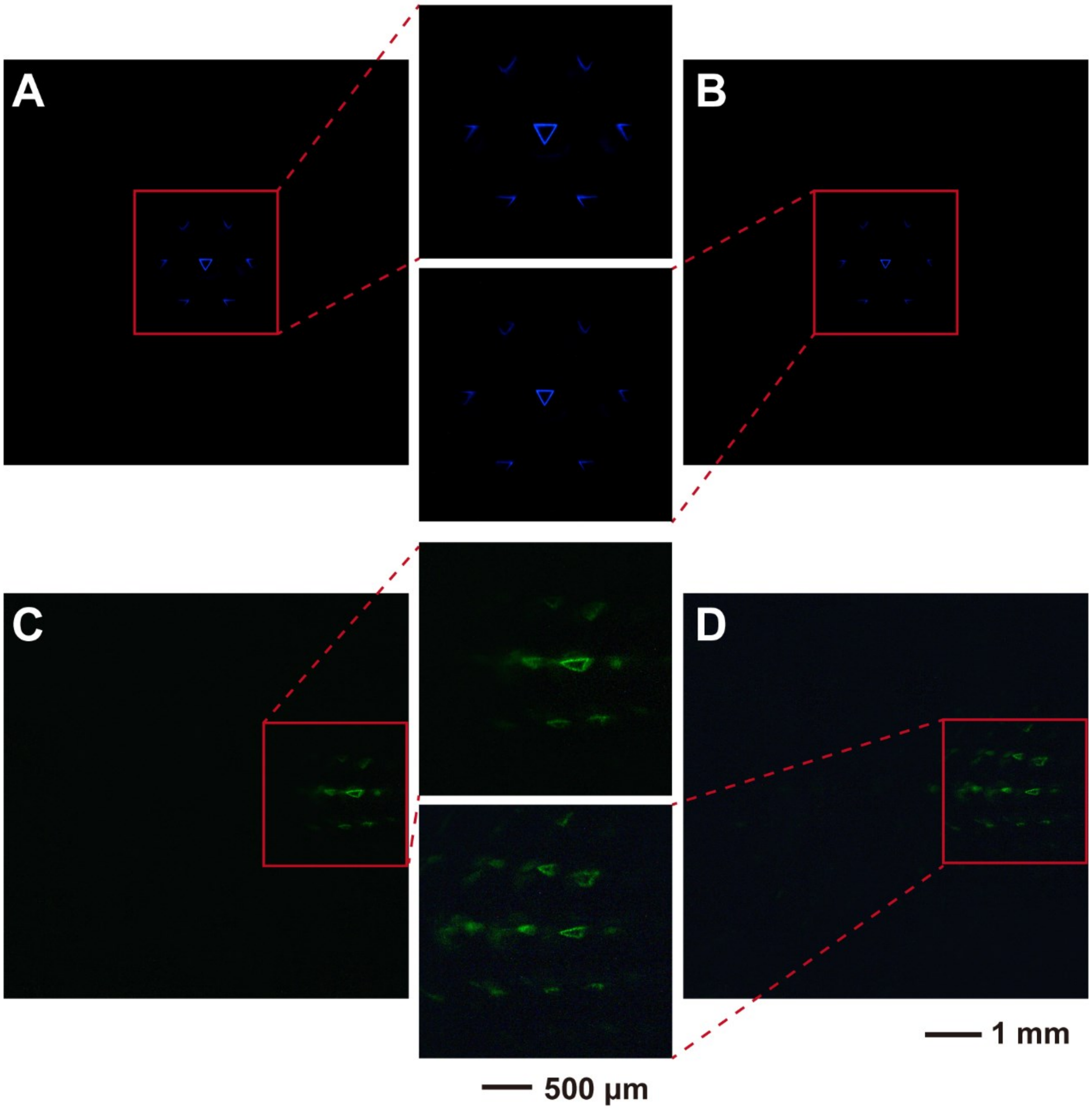

Fig. S12. The demonstration of the imaging using a triangular pattern with a line width of 40 µm. (A) and (B) The triangular pattern was illuminated by blue light and placed in front of the BCE-camera at a distance of 18.9 mm and 42.5 mm, respectively. **(C) and (D)** The triangular pattern was illuminated by green light and placed at the orientation of (α=68.7°, β=0°) with a distance of 19.9 mm and 32.5 mm, respectively.

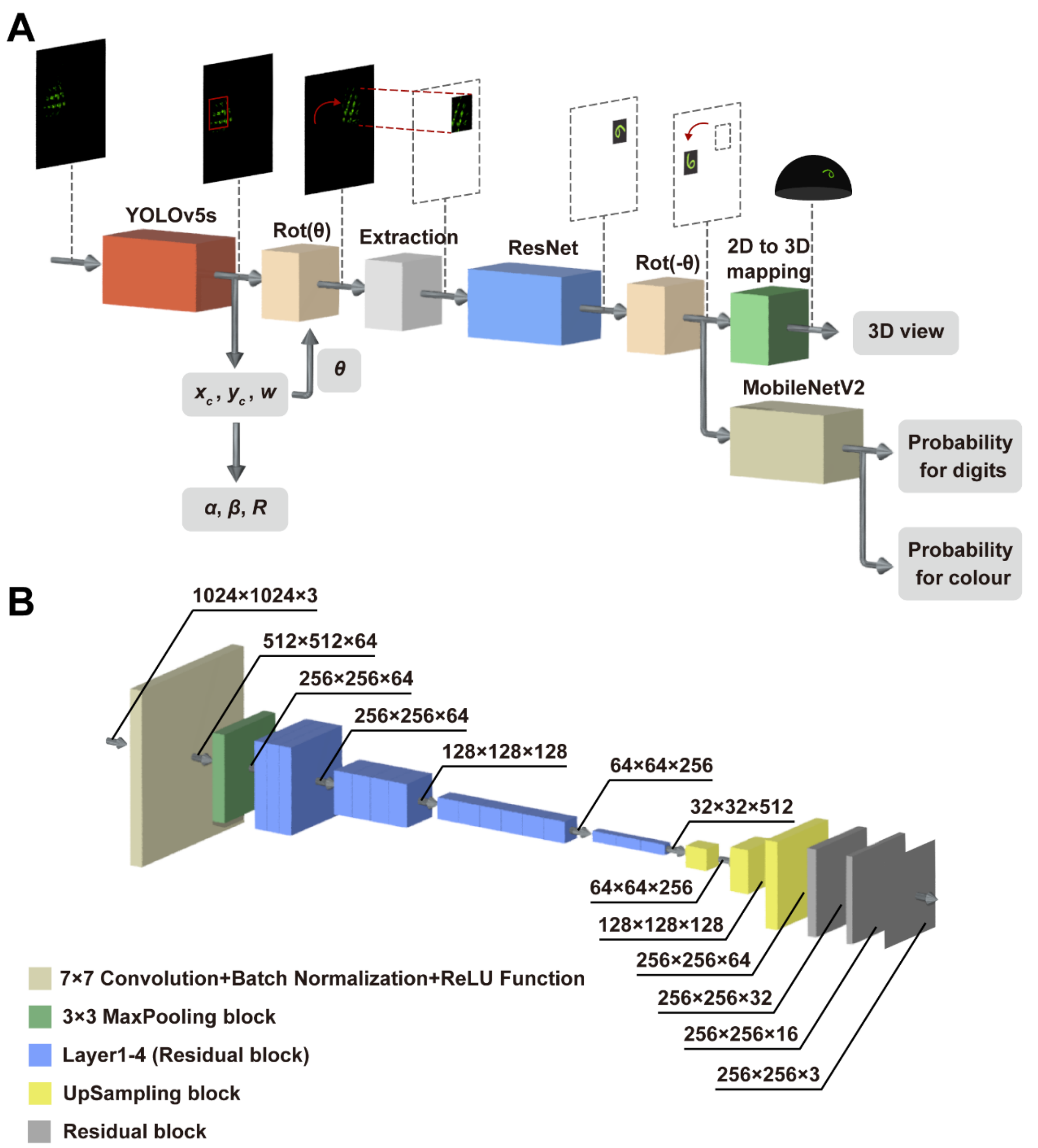

Fig. S13. The multi-stage multi-task neural network. (A) The architecture of the neural network. **(B)** The architecture of the residual neural network.

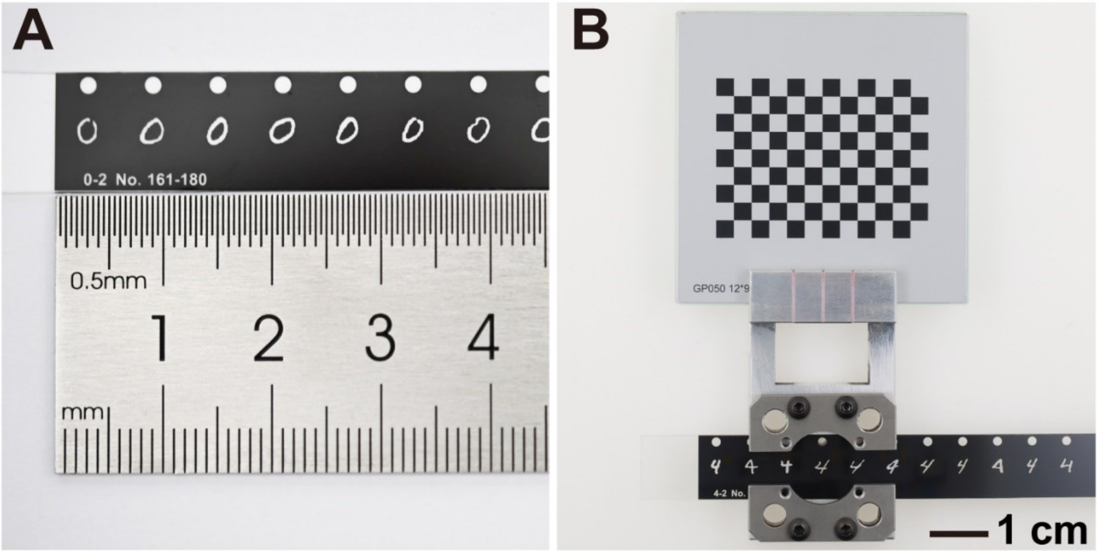

Fig. S14. The mask and the checkerboard pattern. (A) The mask with the handwritten digit pattern ‘0’. **(B)** The mask with the handwritten digit pattern ‘4’ and the checkerboard pattern mounted on the top of the mask.

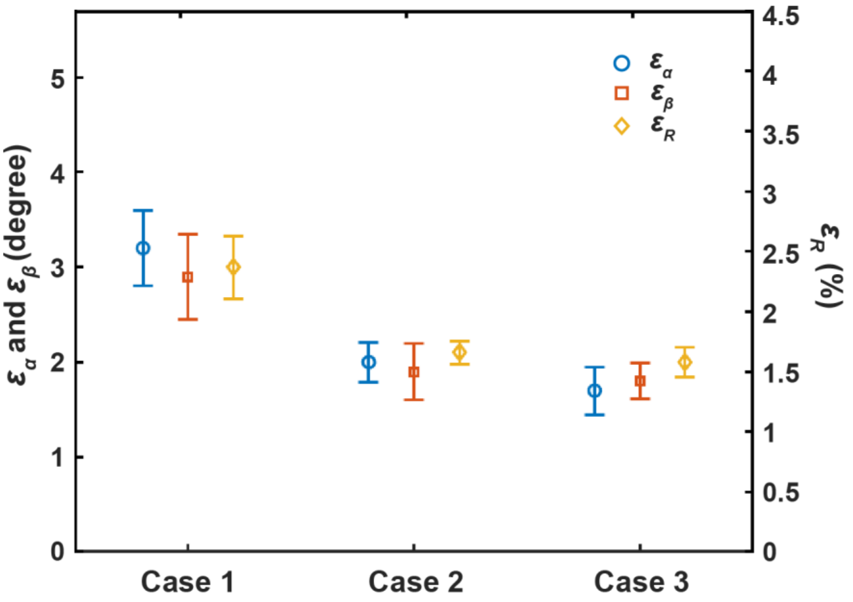

Fig. S15. Influence of the number of positions used for training over the accuracy of 3D positioning. Case 1: 5600 images were collected at 26 positions. Case 2: 6600 images were collected at 32 positions. Case 3: 7700 images were collected at 38 positions. 800 images, including geometry, alphabet and insect patterns, collected at 22 positions were tested, among which 14, 10 and 4 positions were not used in the training for Cases 1, 2 and 3, respectively.

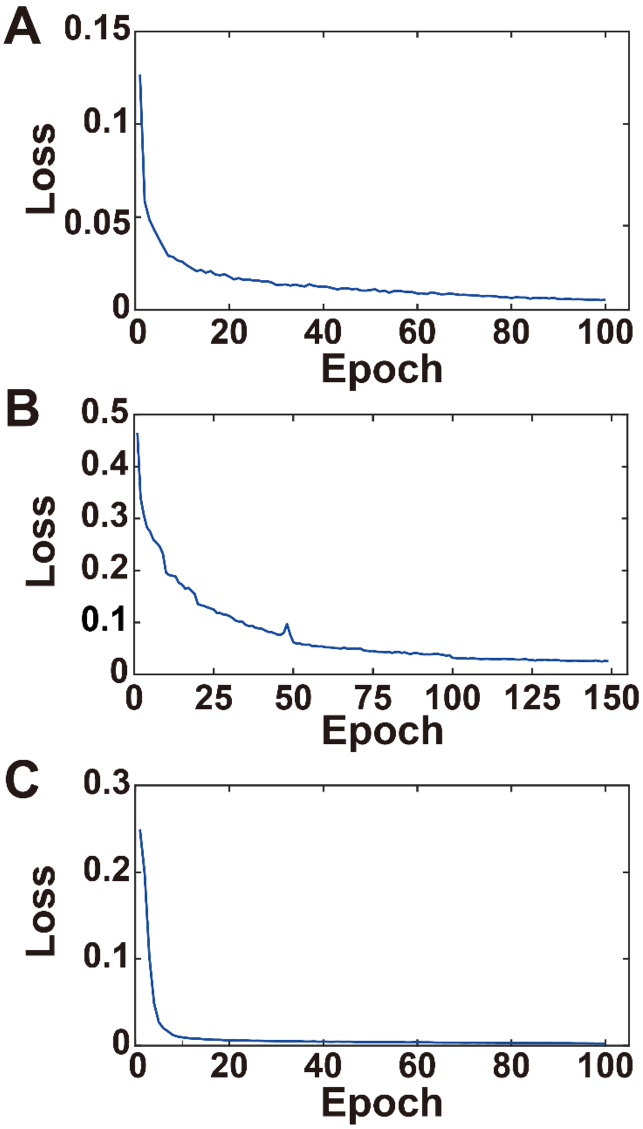

Fig. S16. The learning curves for the neural-network training. (A) The learning curve of the YOLOv5s model for position prediction. The final model was selected around the 90th epoch. **(B)** The learning curve for the neural network for image reconstruction. The final model was selected around the 130th epoch. **(C)** The learning curve for the MobileNetV2 model for digit recognition and color classification. The final model was selected around the 80th epoch.

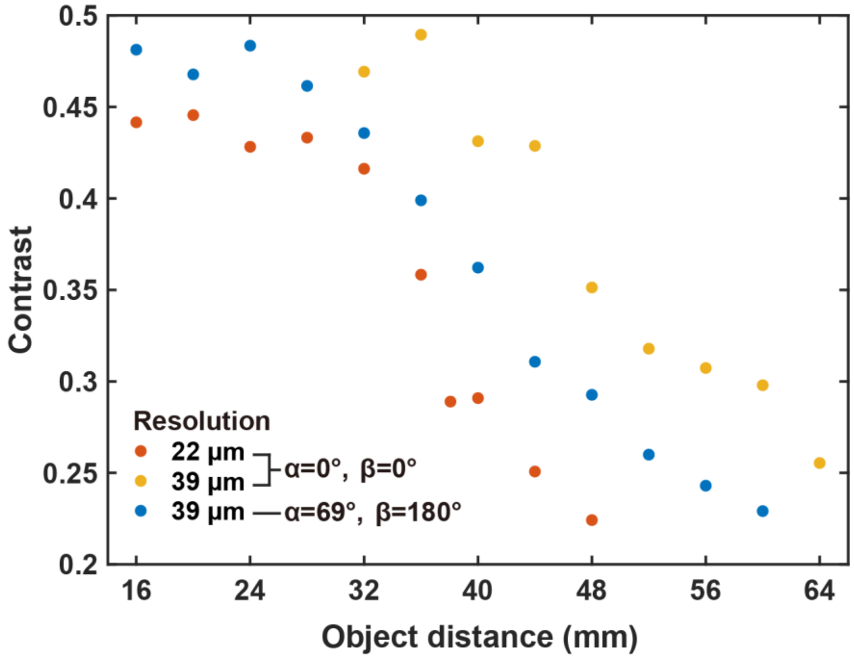

Fig. S17. Influence of the object distance over the image quality. Contrast is defined as $C=(I_{Peak}–I_{Valley})/(I_{Peak}+I_{Valley})$, where I_{Peak} and I_{Valley} are the average peak value and the average valley value for the intensity profile of the line pairs in the resolution target.

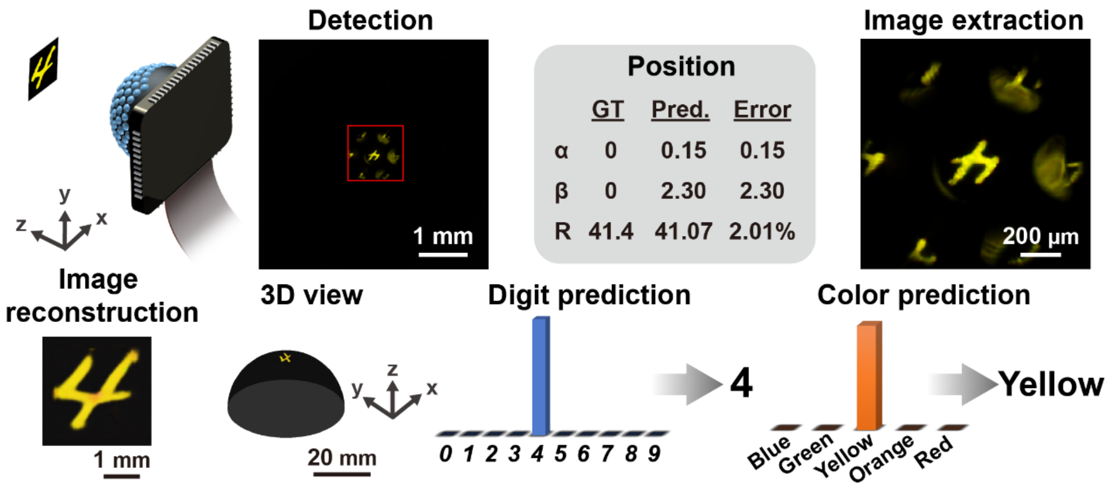

Fig. S18. Panoramic imaging and artificial visual cognition using the BCE-camera. Imaging and recognition of the yellow handwritten digit ‘4’ placed in front of the BCE-camera.

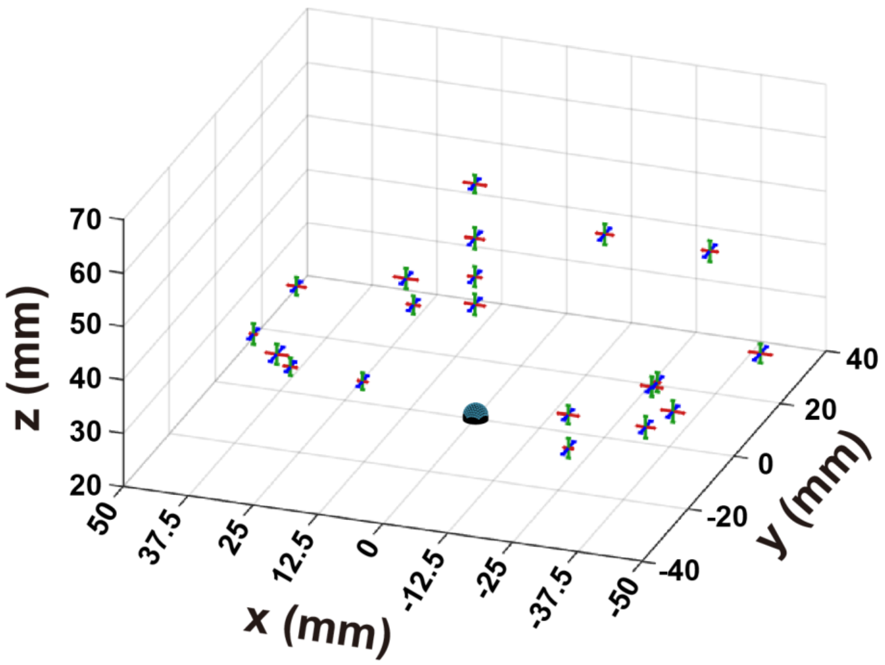

Fig. S19. Errors of the position prediction for testing datasets. There are a total of 800 images, including digit, geometry, alphabet and insect patterns for testing. Red, blue and green error bars were calculated based on the 3D position prediction for the corresponding x, y and z positions, respectively.

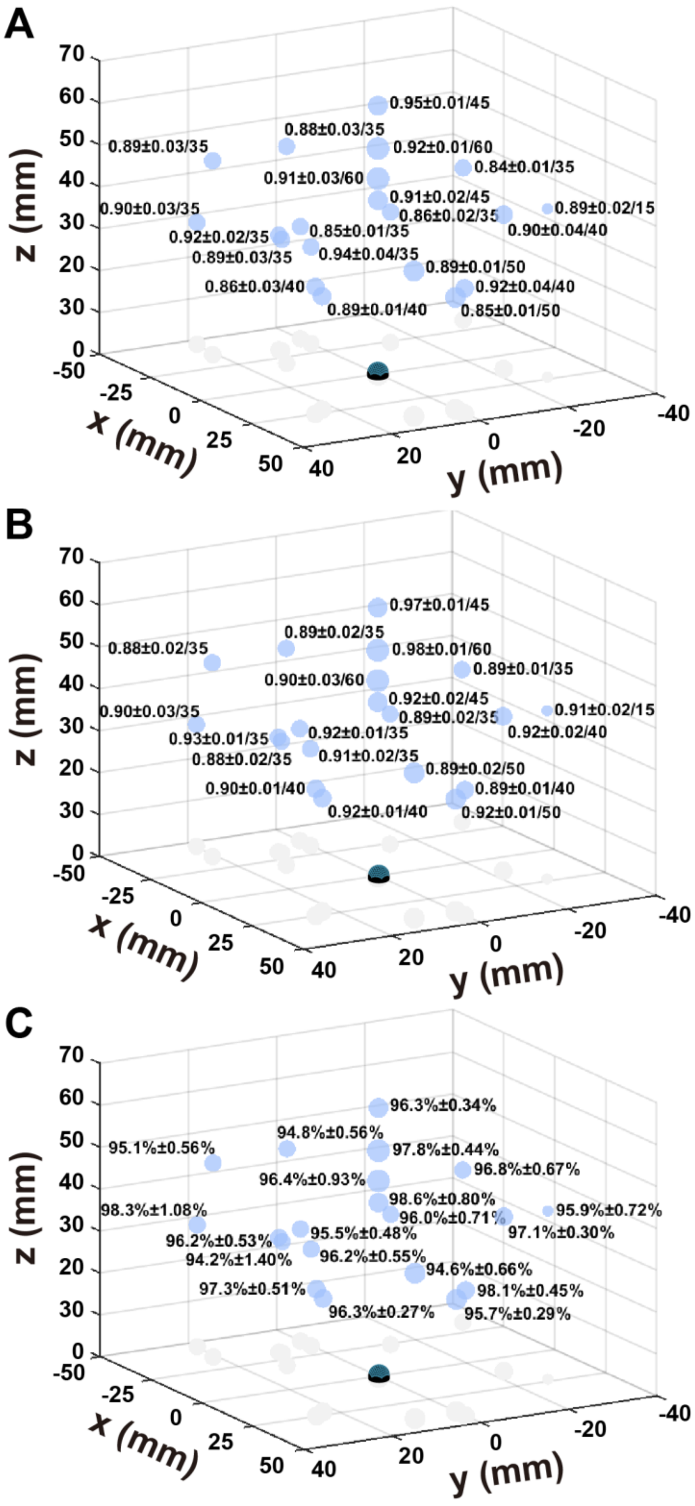

Fig. S20. Evaluation of the reconstructed images. (A) Structural similarity. **(B)** 2D correlation. **(C)** Color similarity. The size of the dots represents the number of testing datasets for the corresponding position. There are a total of 800 images including digit, geometry, alphabet and insect patterns for testing. The number next to the dots is the mean ± standard deviation/the number of the testing dataset for the corresponding position.

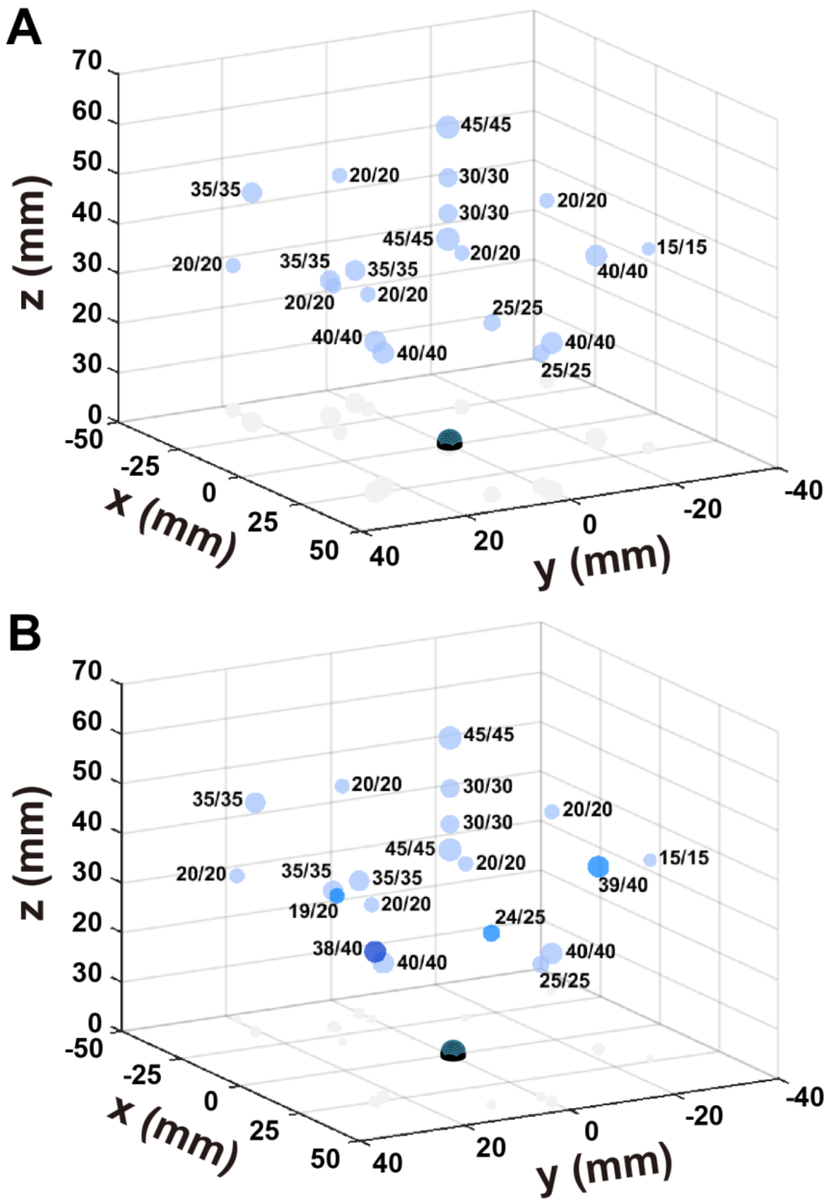

Fig. S21. Statistics on (A) color classification and (B) digit recognition. The size of the dots represents the number of testing datasets for the corresponding position. There are a total of 600 images of digit patterns for testing. The number next to the dots is the number of the correct results/the total number of the testing dataset for the corresponding position. The tint of the color illustrates the accuracy of the prediction.

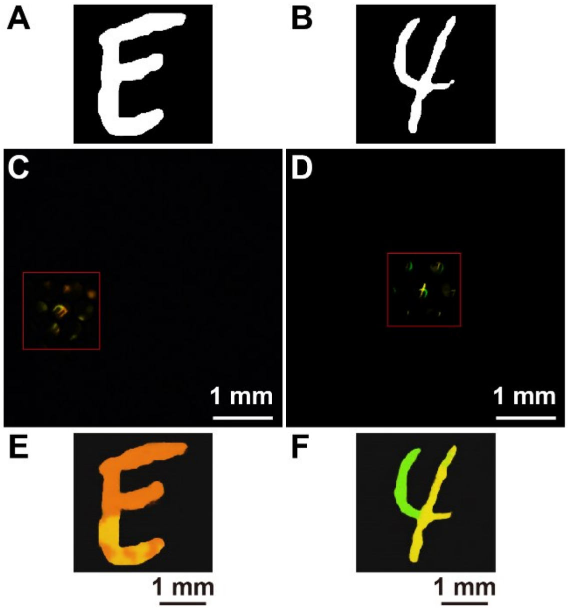

Fig. S22. Detection of two-color patterns using the biomimetic visual system. (A) Patterns of alphabet 'E' and **(B)** digit '4' were used in the demonstration. Colored polyvinyl chloride films were attached to the masks to filter the light. **(C)** and **(D)** The detection images. **(E)** and **(F)** The reconstructed two-color patterns.

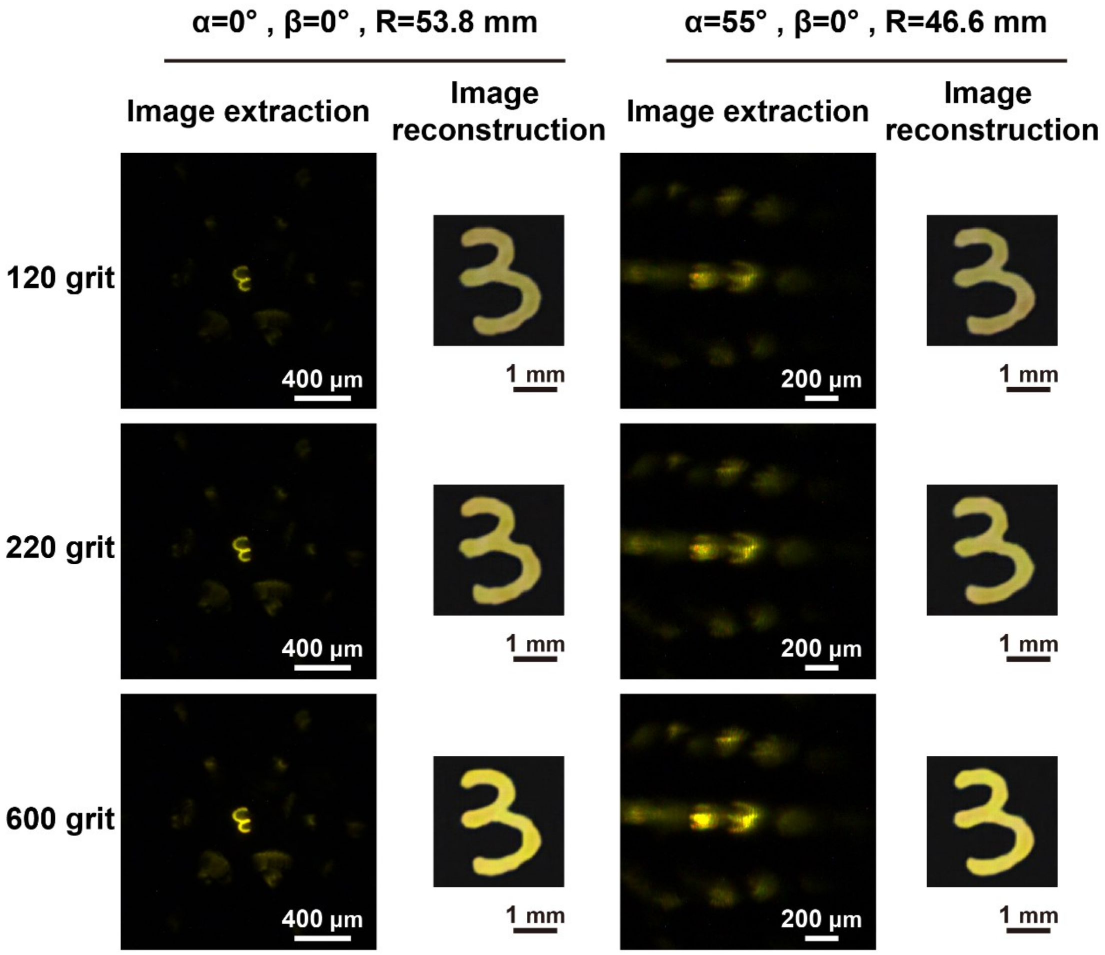

Fig. S23. Detection and reconstruction of the images obtained by the BCE-camera when the illumination light is diffused in different degrees. The illumination light used in the neural network training was generated by a diffuser of 600 grit.

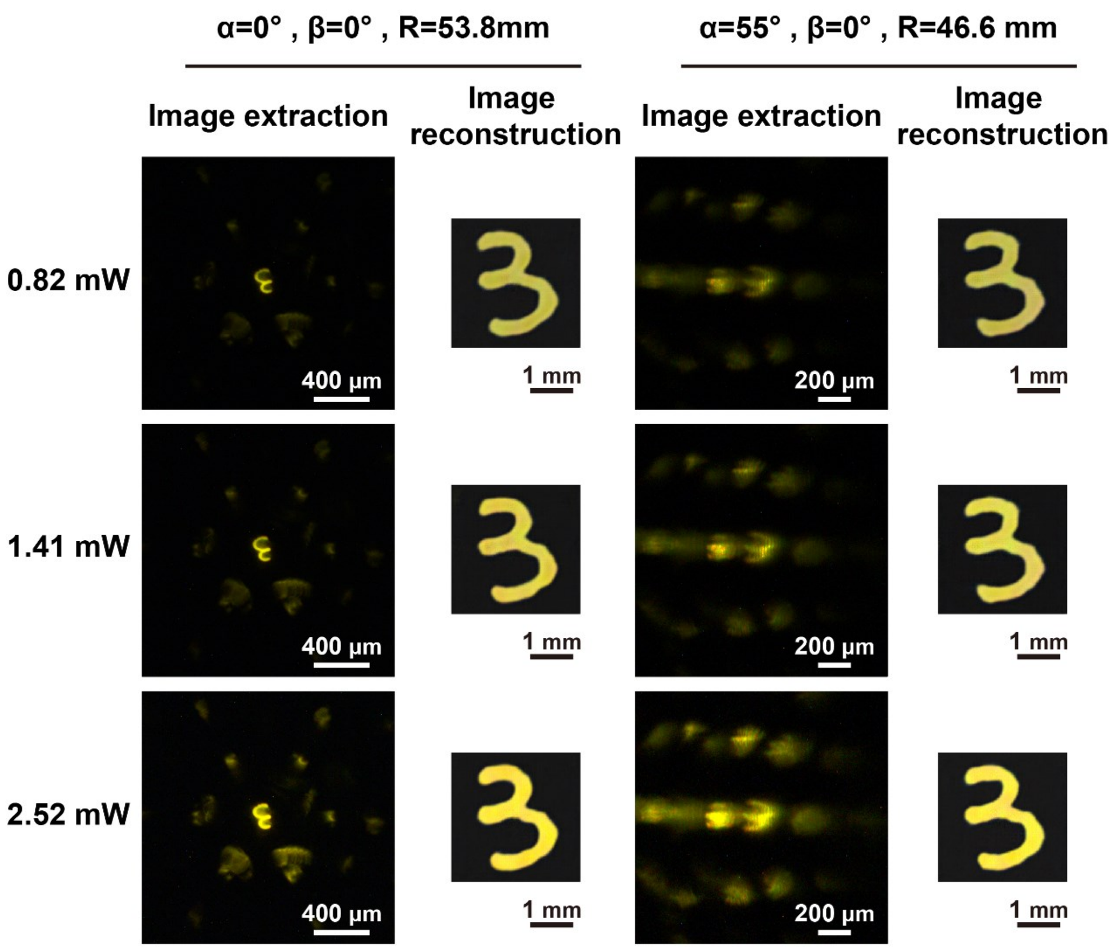

Fig. S24. Detection and reconstruction of the images obtained by the BCE-camera using the illumination light of different intensities. The illumination light used in the neural network training had the optical powers of 2.52 mW for yellow light, 2.76 mW for orange light, 2.43 mW for blue light, 3.03 mW for green light and 2.88 mW for red light, respectively.

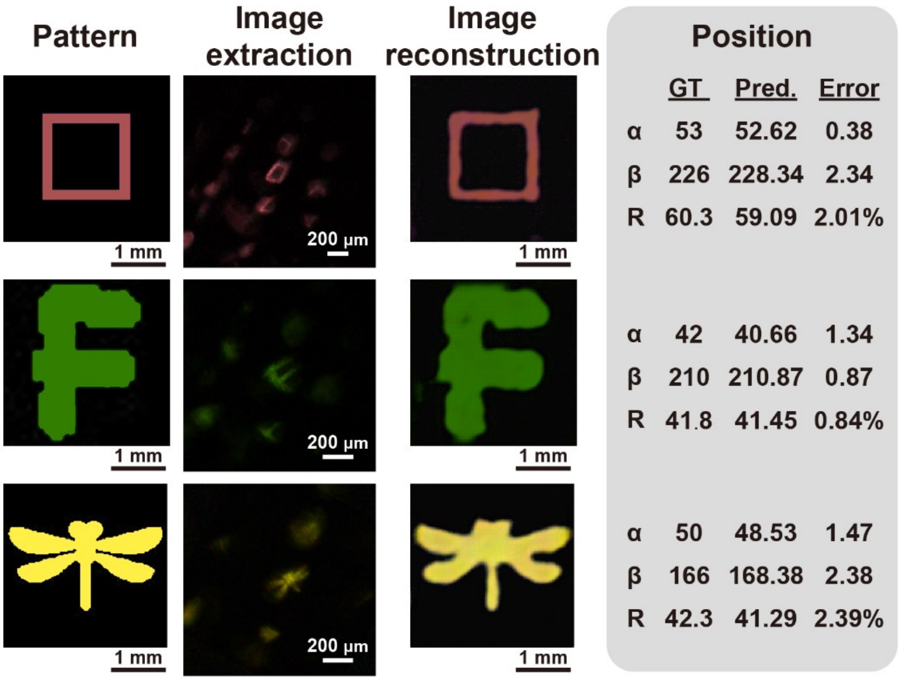

Fig. S25. Detection and reconstruction of the images for different patterns obtained by the BCE-camera at different positions and under different illumination intensities.

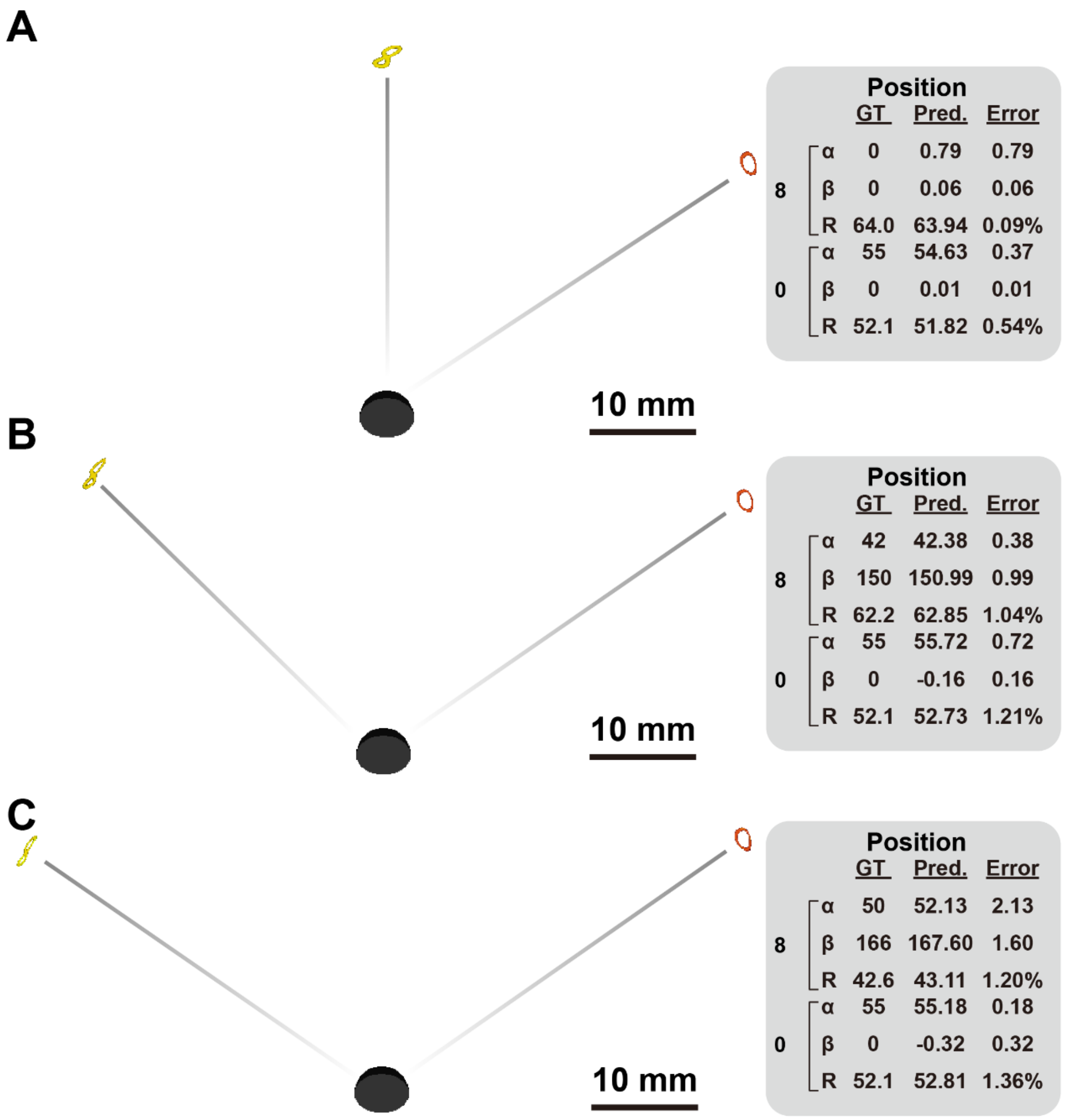

Fig. S26. The 3D views of digit patterns in the demonstration of 3D tracking and imaging. (A-C) correspond to the results presented in **Fig. 6F-H**, respectively.

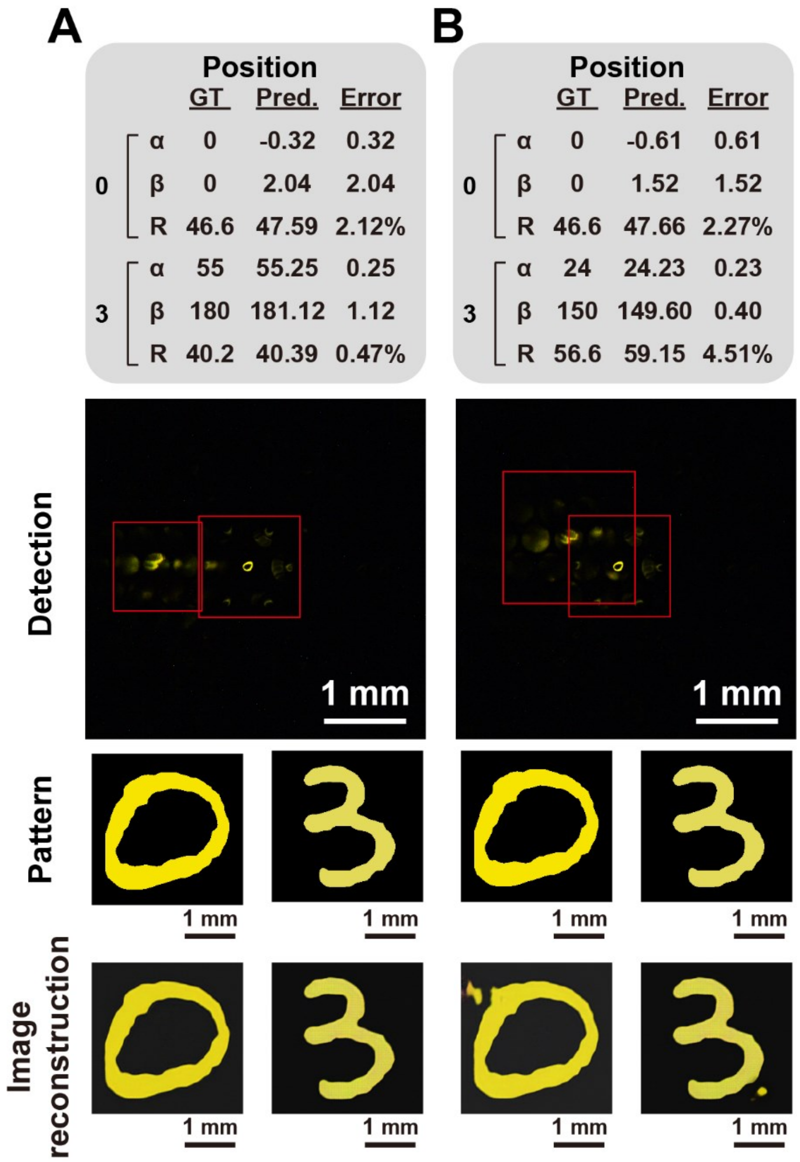

Fig. S27. Investigation of the 3D positioning and image reconstruction capabilities of the biomimetic visual system for detecting two objects in the same colour. The two digit patterns '0' and '3' are closely located. **(A)** The salient regions for the two patterns are partially overlapped. **(B)** The salient regions for the two patterns are severely overlapped.

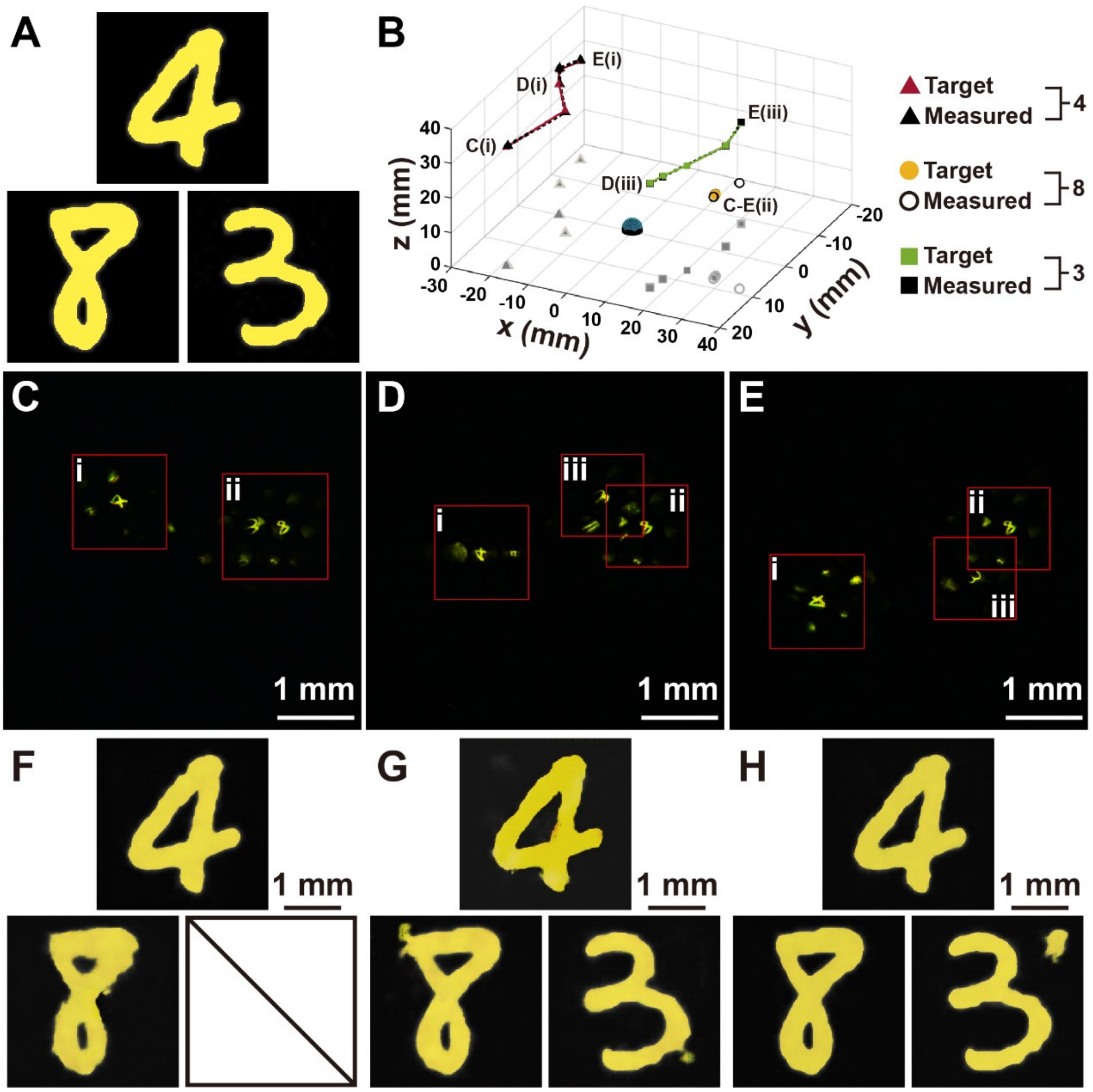

Fig. S28. Demonstration of 3D positioning and imaging for the scenarios involving multiple moving objects. (A) The patterns used in the experiment. **(B)** The spatial positions and the movement paths of the three digits. **(C)-(E)** The images captured by the BCE-camera. **(F)-(H)** The reconstructed digit patterns.

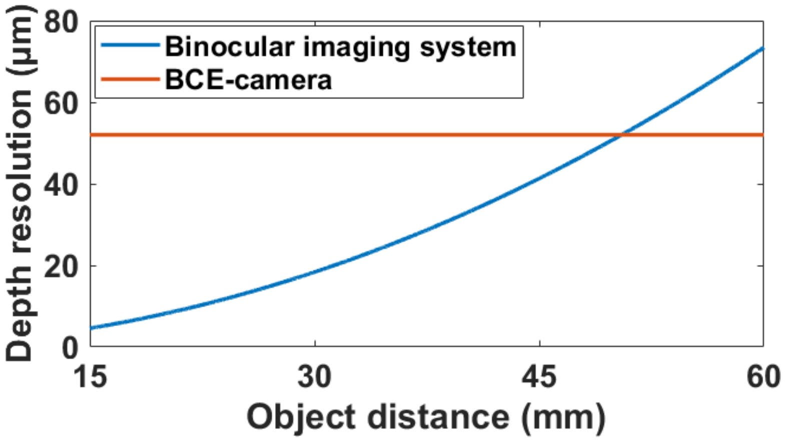

Fig. S29. The depth resolution of the binocular imaging system and the BCE camera. The depth resolution of the binocular imaging system is calculated as $S_Z=u^2S_{Pixel}/bf$, where u is the object distance, S_{Pixel} is the pixel size of the image sensor, b is the baseline distance, and f is the focal length of the lenses. The depth resolution of the BCE-camera is determined by the scale factor, p, for conversion between the pixel number of the tracking box and the distance, R.

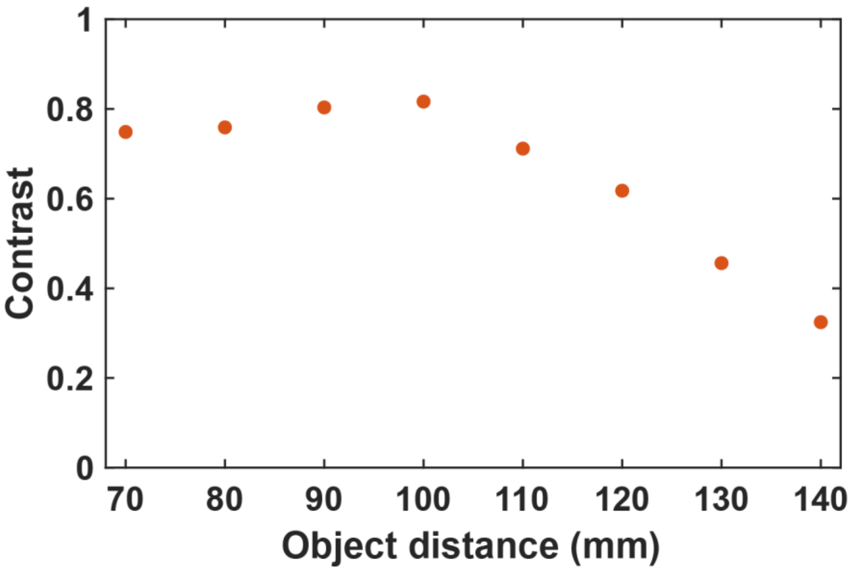

Fig. S30. Spatial resolution of the binocular imaging system. The contrast of the 198.4 μm lines detected by the binocular imaging system with the change of the object distance that covers the detection range of the BCE-camera in the demonstration.

Table S1 Statistics on accuracy of the color classification and pattern recognition.

Pattern	Color classification	Pattern recognition
Digit ‘0’	100%	100%
Digit ‘1’	100%	98.3%
Digit ‘2’	100%	100%
Digit ‘3’	100%	100%
Digit ‘4’	100%	100%
Digit ‘5’	100%	100%
Digit ‘6’	100%	98.3%
Digit ‘7’	100%	96.7%
Digit ‘8’	100%	100%
Digit ‘9’	100%	98.3%
Alphabet ‘A’	100%	98.2%
Alphabet ‘B’	100%	91.8%
Alphabet ‘C’	100%	93.8%
Alphabet ‘D’	100%	94.0%
Alphabet ‘E’	100%	98.5%

Table S2 Performance evaluation when two masks of digit patterns are placed with the minimum separation angle at different distance to the BCE-camera. 20 pairs of digit patterns were tested at each position. The minimum separation angle, $\Delta\alpha_{min}$, is determined by the distance of the masks to the BCE-camera and the size of the masks (7 mm × 7 mm).

**Positions of the objects/ separation angle, $\Delta\alpha_{min}$**	**Prediction errors in 3D positioning, ε_α, ε_β, ε_R**	**Structural similarity, *SSIM* ***	**2D correlation, *corr2D* ***	**Color similarity, *CS* ***	**Color classification ****	**Digit recognition ****
α_1=0° α_2=25° β=0° R=16 mm/ $\Delta\alpha_{min}$=25°	$\varepsilon_{\alpha 1}$=0.12° $\varepsilon_{\beta 1}$=1.05° ε_{R1}= 1.08% $\varepsilon_{\alpha 2}$=0.38° $\varepsilon_{\beta 2}$=1.67° ε_{R2}=0.97%	0.93±0.03/ 0.89±0.02	0.97±0.01/ 0.90±0.01	98.3%±0.46%/ 97.8%±0.27%	20/20	20/20
α_1=0° α_2=16° β=19° R=26 mm/ $\Delta\alpha_{min}$=16°	$\varepsilon_{\alpha 1}$=0.57° $\varepsilon_{\beta 1}$=1.23° ε_{R1}=1.17% $\varepsilon_{\alpha 2}$=0.99° $\varepsilon_{\beta 2}$=1.10° ε_{R2}=1.13%	0.93±0.02/ 0.88±0.03	0.91±0.03/ 0.89±0.02	97.5%±0.28%/ 96.3%±0.52%	20/20	20/20
α_1=26° α_2=46° β=120° R=20 mm/ $\Delta\alpha_{min}$=20°	$\varepsilon_{\alpha 1}$=0.16° $\varepsilon_{\beta 1}$=1.22° ε_{R1}=1.30% $\varepsilon_{\alpha 2}$=0.82° $\varepsilon_{\beta 2}$=2.05° ε_{R2}=1.29%	0.90±0.01/ 0.89±0.03	0.92±0.01/ 0.94±0.01	95.9%±1.08%/ 95.2%±1.40%	20/20	20/20
α_1=10° α_2=23° β=230° R=33 mm/ $\Delta\alpha_{min}$=13°	-	-	-	-	-	-
α_1=35° α_2=50° β=350° R=28 mm/ $\Delta\alpha_{min}$=15°	$\varepsilon_{\alpha 1}$=0.13° $\varepsilon_{\beta 1}$=1.93° ε_{R1}=1.45% $\varepsilon_{\alpha 2}$=0.64° $\varepsilon_{\beta 2}$=1.22° ε_{R2}=2.25%	0.88±0.03/ 0.87±0.04	0.89±0.02/ 0.88±0.01	96.8%±0.29%/ 94.8%±0.71%	20/20	20/19

* The mean ± standard deviation for Object 1/the mean ± standard deviation for Object 2

** The number of the correct results for Object 1/the number of the correct results for Object 2

- indicates that some testing fails

Table S3 Comparison of the state-of-the-art biomimetic compound eye based visual systems.

Imaging sensor	Scalability*	Full color imaging	AOV (°)	Spatial resolution (μm)	Number of effective pixels (pixel)	Function	Ref.
Hemispherical perovskite nanowire photodetector array	625<br>20 mm<br>1 mm	No	140°/220°<br>(single/pair)	Not given	1,600	● Panoramic imaging<br>● Motion detection	14
Silicon photodetector	630<br>128 mm<br>172 μm	No	180°/60°<br>(horizontal/ vertical)	Not given	630	● Panoramic imaging	19
Silicon photodiodes	180<br>>10 mm<br>800 μm	No	160°	Not given	180	● Panoramic imaging	20
Flexible comb-shaped silicon photodiode	256<br>~25 mm<br>400 μm	No	300°×160°	Not given	256	● Panoramic imaging<br>● Imaging in both air and water	21
Complementary metal oxide semiconductor image sensor	160<br>400 μm<br>40 μm	Yes	90°	Not given	80,000	● Panoramic imaging<br>● 3D positioning<br>● Motion detection	23
Complementary metal oxide semiconductor image sensor	522<br>5 mm<br>180 μm	Yes	170°	Not given	522	● 3D positioning<br>● Panoramic imaging<br>● Motion detection	24
Complementary metal oxide semiconductor image sensor	271<br>14 mm<br>250 μm	Yes	180°	Not given	271	● 3D positioning<br>● Panoramic imaging<br>● Motion detection	49
InGaAs photodiodes	256<br>40 mm<br>300 μm	No	160°	Not given	512<br>(pair)	● 3D positioning<br>● Motion detection	53
Complementary metal oxide semiconductor image sensor	127<br>5 mm<br>455 μm	Yes	165°	40 μm	4,251,325	● 3D positioning<br>● Panoramic imaging<br>● Multi-target tracking<br>● Pattern recognition<br>● Color classification	This work

* Scalability: number of ommatidia / diameter of eye (mm) / size of each ommatidium

REFERENCES AND NOTES

1. C. Aria, J.-B. Caron, A middle Cambrian arthropod with chelicerae and proto-book gills. *Nature* **573**, 586–589 (2019).

2. N. J. Strausfeld, X. Ma, G. D. Edgecombe, Fossils and the evolution of the arthropod brain. *Curr. Biol.* **26**, R989–R1000 (2016).

3. D.-E. Nilsson, A. Kelber, A functional analysis of compound eye evolution. *Arthropod Struct. Dev.* **36**, 373–385 (2002).

4. J. Vannier, B. Schoenemann, T. Gillot, S. Charbonnier, E. Clarkson, Exceptional preservation of eye structure in arthropod visual predators from the Middle Jurassic. *Nat. Commun.* **7**, 10320 (2016).

5. J. P. Currea, Y. Sondhi, A. Y. Kawahara, J. Theobald, Measuring compound eye optics with microscope and microCT images. *Commun. Biol.* **6**, 246 (2023).

6. A. Avarguès-Weber, N. Deisig, M. Giurfa, Visual cognition in social insects. *Annu. Rev. Entomol.* **56**, 423–443 (2011).

7. K. H. Jeong, J. Kim, L. P. Lee, Biologically inspired artificial compound eyes. *Science* **312**, 557–561 (2006).

8. D. Keum, K.-W. Jang, D. S. Jeon, C. S. H. Hwang, E. K. Buschbeck, M. H. Kim, K.-H. Jeong, Xenos peckii vision inspires an ultrathin digital camera. *Light Sci. Appl.* **7**, 80 (2018).

9. J.-J. Kim, H. Liu, A. O. Ashtiani, H. Jiang, Biologically inspired artificial eyes and photonics. *Rep. Prog. Phys.* **83**, 047101 (2020).

10. H. Liu, Y. Huang, H. Jiang, Artificial eye for scotopic vision with bioinspired all-optical photosensitivity enhancer. *Proc. Natl. Acad. Sci. U.S.A.* **113**, 3982–3985 (2016).

11. C.-C. Huang, X. Wu, H. Liu, B. Aldalali, J. A. Rogers, H. Jiang, Large-field-of-view wide-spectrum artificial reflecting superposition compound eyes. *Small* **10**, 3050–3057 (2014).

12. Z. Deng, F. Chen, Q. Yang, H. Bian, G. Du, J. Yong, C. Shan, X. Hou, Dragonfly-eye-inspired artificial compound eyes with sophisticated imaging. *Adv. Funct. Mater.* **26**, 1995–2001 (2016).

13. Z. Rao, Y. Lu, Z. Li, K. Sim, Z. Ma, J. Xiao, C. Yu, Curvy, shape-adaptive imagers based on printed optoelectronic pixels with a kirigami design. *Nat. Electron.* **4**, 513–521 (2021).

14. Y. Zhou, Z. Sun, Y. Ding, Z. Yuan, X. Qiu, Y. B. Cao, Z. Wan, Z. Long, S. Poddar, S. Kumar, W. Ye, C. L. J. Chan, D. Zhang, B. Ren, Q. Zhang, H.-S. Kwok, M. G. Li, Z. Fan, An ultrawide field-of-view pinhole compound eye using hemispherical nanowire array for robot vision. *Sci. Robot.* **9**, eadi8666 (2024).

15. M. Wu, Z. Ma, Z. Tian, J. T. Rich, X. He, J. Xia, Y. He, K. Yang, S. Yang, K. W. Leong, L. P. Lee, T. J. Huang, Sound innovations for biofabrication and tissue engineering. *Microsyst. Nanoeng.* **10**, 170 (2024).

16. L. Gu, S. Poddar, Y. Lin, Z. Long, D. Zhang, Q. Zhang, L. Shu, X. Qiu, M. Kam, A. Javey, Z. Fan, A biomimetic eye with a hemispherical perovskite nanowire array retina. *Nature* **581**, 278–282 (2020).

17. H. Jiang, Artificial eye boosted by hemispherical retina. *Nature* **581**, 264–265 (2020).

18. Y. Ding, G. Liu, Z. Long, Y. Zhou, X. Qiu, B. Ren, Q. Zhang, C. Chi, Z. Wan, B. Huang, Z. Fan, Uncooled self-powered hemispherical biomimetic pit organ for mid- to long-infrared imaging. *Sci. Adv.* **8**, eabq8432 (2022).

19. D. Floreano, R. Pericet-Camara, S. Viollet, F. Ruffier, A. Brückner, R. Leitel, W. Buss, M. Menouni, F. Expert, R. Juston, M. K. Dobrzynski, G. L’Eplattenier, F. Recktenwald, H. A. Mallot, N. Franceschini, Miniature curved artificial compound eyes. *Proc. Natl. Acad. Sci. U.S.A.* **110**, 9267–9272 (2013).

20. Y. M. Song, Y. Xie, V. Malyarchuk, J. Xiao, I. Jung, K.-J. Choi, Z. Liu, H. Park, C. Lu, R.-H. Kim, R. Li, K. B. Crozier, Y. Huang, J. A. Rogers, Digital cameras with designs inspired by the arthropod eye. *Nature* **497**, 95–99 (2013).

21. M. Lee, G. J. Lee, H. J. Jang, E. Joh, H. Cho, M. S. Kim, H. M. Kim, K. M. Kang, J. H. Lee, M. Kim, H. Jang, J.-E. Yeo, F. Durand, N. Lu, D.-H. Kim, Y. M. Song, An amphibious artificial vision system with a panoramic visual field. *Nat. Electron.* **5**, 452–459 (2022).

22. M. Kim, S. Chang, M. Kim, J.-E. Yeo, M. S. Kim, G. J. Lee, D.-H. Kim, Y. M. Song, Cuttlefish eye–inspired artificial vision for high-quality imaging under uneven illumination conditions. *Sci. Robot.* **8**, eade4698 (2023).

23. Z.-Y. Hu, Y.-L. Zhang, C. Pan, J.-Y. Dou, Z.-Z. Li, Z.-N. Tian, J.-W. Mao, Q.-D. Chen, H.-B. Sun, Miniature optoelectronic compound eye camera. *Nat. Commun.* **13**, 5634 (2022).

24. B. Dai, L. Zhang, C. Zhao, H. Bachman, R. Becker, J. Mai, Z. Jiao, W. Li, L. Zheng, X. Wan, T. J. Huang, S. Zhuang, D. Zhang, Biomimetic apposition compound eye fabricated using microfluidic-assisted 3D printing. *Nat. Commun.* **12**, 6458 (2021).

25. Z. Zhang, S. Wang, C. Liu, R. Xie, W. Hu, P. Zhou, All-in-one two-dimensional retinomorphic hardware device for motion detection and recognition. *Nat. Nanotechnol.* **17,** 27–32 (2022).

26. F. Liao, Z. Zhou, B. J. Kim, J. Chen, J. Wang, T. Wan, Y. Zhou, A. T. Hoang, C. Wang, J. Kang, J.-H. Ahn, Y. Chai, Bioinspired in-sensor visual adaptation for accurate perception. *Nat. Electron.* **5**, 84–91 (2022).

27. P. Huang, B. Jiang, H. Chen, J. Xu, K. Wang, C. Zhu, X. Hu, D. Li, L. Zhen, F. Zhou, J. Qin, C. Xu, Neuro-inspired optical sensor array for high-accuracy static image recognition and dynamic trace extraction. *Nat. Commun.* **14**, 6736 (2023).

28. X. Feng, C. Li, J. Song, Y. He, W. Qu, W. Li, K. Guo, L. Liu, B. Yang, H. Wei, Differential perovskite hemispherical photodetector for intelligent imaging and location tracking. *Nat. Commun.* **15**, 577 (2024).

29. J. Chen, Z. Zhou, B. J. Kim, Y. Zhou, Z. Wang, T. Wan, J. Yan, J. Kang, J.-H. Ahn, Y. Chai, Optoelectronic graded neurons for bioinspired in-sensor motion perception. *Nat. Nanotechnol.* **18**, 882–888 (2023).

30. Y. Wang, Y. Gong, S. Huang, X. Xing, Z. Lv, J. Wang, J.-Q. Yang, G. Zhang, Y. Zhou, S.-T. Han, Memristor-based biomimetic compound eye for real-time collision detection. *Nat. Commun.* **12**, 5979 (2021).

31. C. Chen, Y. He, H. Mao, L. Zhu, X. Wang, Y. Zhu, Y. Zhu, Y. Shi, C. Wan, Q. Wan, A photoelectric spiking neuron for visual depth perception. *Adv. Mater.* **34**, e2201895 (2022).

32. J. Redmon, S. Divvala, R. Girshick, A. Farhadi, "You only look once: Unified, real-time object detection," in *Proceedings of the IEEE Conference on Computer Vision and Pattern Recognition* (IEEE, 2016), pp. 779–788.

33. L. Huang, Q. Fu, M. He, D. Jiang, Z. Hao, Detection algorithm of safety helmet wearing based on deep learning. *Concurr. Comput.* **33**, e6234 (2021).

34. J. Fang, A. Swain, R. Unni, Y. Zheng, Decoding optical data with machine learning. *Laser Photon. Rev.* **15**, 2000422 (2021).

35. A. D. C. Fonseca, C. Glück, J. Droux, Y. Ferry, C. Frei, S. Wegener, B. Weber, M. E. Amki, D. Ahmed, Ultrasound trapping and navigation of microrobots in the mouse brain vasculature. *Nat. Commun.* **14**, 5889 (2023).

36. J. Rufo, F. Cai, J. Friend, M. Wiklund, T. J. Huang, Acoustofluidics for biomedical applications. *Nat. Rev. Methods Primers* **2**, 30 (2022).

37. D. Ahmed, A. Sukhov, D. Hauri, D. Rodrigue, G. Maranta, J. Harting, B. J. Nelson, Bioinspired acousto-magnetic microswarm robots with upstream motility. *Nat. Mach. Intell.* **3**, 116–124 (2021).

38. S. H. Hilton, I. M. White, Advances in the analysis of single extracellular vesicles: A critical review. *Sens. Actuators Rep.* **3**, 100052 (2021).

39. J. Rufo, P. Zhang, R. Zhong, L. P. Lee, T. J. Huang, A sound approach to advancing healthcare systems: The future of biomedical acoustics. *Nat. Commun.* **13**, 3459 (2022).

40. R. Snodgrass, A. Gardner, A. Semeere, V. L. Kopparthy, J. Duru, T. Maurer, J. Martin, E. Cesarman, D. Erickson, A portable device for nucleic acid quantification powered by sunlight, a flame or electricity. *Nat. Biomed. Eng.* **2**, 657–665 (2018).

41. K. Kim, K.-W. Jang, J.-K. Ryu, K.-H. Jeong, Biologically inspired ultrathin arrayed camera for high-contrast and high-resolution imaging. *Light Sci. Appl.* **9**, 28 (2020).

42. S.-I. Bae, K. Kim, K.-W. Jang, H.-K. Kim, K.-H. Jeong, High contrast ultrathin light-field camera using inverted microlens arrays with metal–insulator–metal optical absorber. *Adv. Opt. Mater.* **9**, 2001657 (2021).

43. P. Wimmer, J. Mehnert, A. P. Condurache, Dimensionality reduced training by pruning and freezing parts of a deep neural network: A survey. *Artif. Intell. Rev.* **56**, 14257–14295 (2023).

44. W. Jia, M. Sun, J. Lian, S. Hou, Feature dimensionality reduction: A review. *Complex Intell. Syst.* **8**, 2663–2693 (2022).

45. Y. Li, J. Li, Y. Zhao, T. Gan, J. Hu, M. Jarrahi, A. Ozcan, Universal polarization transformations: Spatial programming of polarization scattering matrices using a deep learning-designed diffractive polarization transformer. *Adv. Mater.* **35**, e2303395 (2023).

46. Z. Zhao, Y. Wang, C. Guan, K. Zhang, Q. Wu, H. Li, J. Liu, S. N. Burokur, X. Ding, Deep learning-enabled compact optical trigonometric operator with metasurface. *PhotoniX* **3**, 15 (2022).

47. Y. Chen, M. Nazhamaiti, H. Xu, Y. Meng, T. Zhou, G. Li, J. Fan, Q. Wei, J. Wu, F. Qiao, L. Fang, Q. Dai, All-analog photoelectronic chip for high-speed vision tasks. *Nature* **623**, 48–57 (2023).

48. H. Cai, Z. Ao, C. Tian, Z. Wu, H. Liu, J. Tchieu, M. Gu, K. Mackie, F. Guo, Brain organoid reservoir computing for artificial intelligence. *Nat. Electron.* **6**, 1032–1039 (2023).

49. H. Jiang, C. C. Tsoi, W. Yu, M. Ma, M. Li, Z. Wang, X. Zhang, Optical fibre based artificial compound eyes for direct static imaging and ultrafast motion detection. *Light Sci. Appl.* **13**, 256 (2024).

50. K. He, X. Zhang, S. Ren, J. Sun, “Deep residual learning for image recognition,” in *Proceedings of the IEEE Conference on Computer Vision and Pattern Recognition* (IEEE, 2016), pp. 770–778.

51. A. G. Howard, M. Zhu, B. Chen, D. Kalenichenko, W. Wang, T. Weyand, M. Andreetto, H. Adam, MobileNets: Efficient convolutional neural networks for mobile vision applications. arXiv:1704.04861 [cs.CV] (2017).

52. B. Dai, S. You, K. Wang, Y. Long, J. Chen, N. Upreti, J. Peng, L. Zheng, C. Chang, T. J. Huang, Y. Guan, S. Zhuang, D. Zhang, Deep learning-enabled filter-free fluorescence microscope. *Sci. Adv.* **11**, eadq2494 (2025).

53. B. Bae, D. Lee, M. Park, Y. Mu, Y. Baek, I. Sim, C. Shen, K. Lee, Stereoscopic artificial compound eyes for spatiotemporal perception in three-dimensional space. *Sci. Robot.* **9**, eadl3606 (2024).
